# A fluorescent paramagnetic Mn metal–organic framework based on semi-rigid pyrene tetra­carboxylic acid: sensing of solvent polarity and explosive nitroaromatics

**DOI:** 10.1107/S2052252515012506

**Published:** 2015-08-14

**Authors:** Alankriti Bajpai, Arindam Mukhopadhyay, Manchugondanahalli Shivakumar Krishna, Savitha Govardhan, Jarugu Narasimha Moorthy

**Affiliations:** a Department of Chemistry, Indian Institute of Technology, Kanpur 208016, India

**Keywords:** crystal structure, metal–organic framework, solvatochromism, fluorescence, sensing, nitro­aromatics

## Abstract

An Mn metal–organic framework (Mn-MOF), Mn-*L*, based on a pyrene-tetraacid linker (H_4_ *L*), displays a respectable fluorescence quantum yield of 8.3% in spite of the presence of the paramagnetic metal ions, due presumably to fixation of the metal ions in geometries that do not allow complete energy/charge-transfer quenching. Remarkably, the porous Mn-*L* MOF with ∼25% solvent-accessible volume exhibits a heretofore unprecedented solvent-dependent fluorescence emission maximum, permitting its use as a probe of solvent polarity; the emission maxima in different solvents correlate excellently with Reichardt’s solvent polarity parameter (*E*_T_^N^). Further, the applicability of Mn-*L* to the sensing of nitroaromatics *via* fluorescence quenching is demonstrated; the detection limit for TNT is shown to be 125 p.p.m. The results bring out the fact that MOFs based on paramagnetic metal ions can indeed find application when the quenching mechanisms are attenuated by certain geometries of the organic linkers of the MOF.

## Abstract 2

Fluorescence quenching by paramagnetic metal ions is attenuated in an Mn metal–organic framework (Mn-MOF) based on a pyrene linker. Based on solvent-dependent emission, the Mn-MOF is shown to serve as a solvent polarity probe. Further, sensing of nitroaromatics is demonstrated with a detection limit of ∼125 p.p.m. for TNT.

## 1. Introduction   

Metal–organic frameworks (MOFs) are a fascinating class of crystalline porous materials, which are being explored intensely for diverse applications such as gas storage (Britt *et al.*, 2008 ▸; Murray *et al.*, 2009 ▸; Makal *et al.*, 2012 ▸; Suh *et al.*, 2012 ▸; Furukawa *et al.*, 2013 ▸), separation (Sumida *et al.*, 2012 ▸; Li *et al.*, 2012 ▸; Wu, Gong *et al.*, 2012 ▸; Wu, Wang *et al.*, 2012 ▸; Nugent *et al.*, 2013 ▸), heterogeneous catalysis (Lee *et al.*, 2009 ▸; Ma *et al.*, 2009 ▸; Yoon *et al.*, 2012 ▸; Moon *et al.*, 2013 ▸), optoelectronics (Wang *et al.*, 2012 ▸; Zhang & Xiong, 2012 ▸), energy storage and conversion (Li *et al.*, 2011 ▸; Shimizu *et al.*, 2013 ▸; Sun *et al.*, 2013 ▸), and drug delivery and bio-imaging (Rocca *et al.*, 2011 ▸; Horcajada *et al.*, 2012 ▸). MOFs with luminescence properties have emerged as appealing materials, due to the fact that they can serve as sensory systems of analytes that are bound to the MOFs by virtue of their porosity (Allendorf *et al.*, 2009 ▸; Lan *et al.*, 2009 ▸; Meek *et al.*, 2011 ▸; Rocha *et al.*, 2011 ▸; Xu *et al.*, 2011 ▸; Cui *et al.*, 2012 ▸; Kreno *et al.*, 2012 ▸; Zhang *et al.*, 2014 ▸; Liu *et al.*, 2015 ▸; Zhang *et al.*, 2015 ▸). Indeed, a new term, LMOFs, has been advanced to refer to such luminescent MOFs (Hu *et al.*, 2014 ▸). In general, luminescence in MOFs arises due to one or more of the following: (i) fluorescent organic linkers; (ii) luminescent metal ions such as lanthanide ions; (iii) a combination of both (i) and (ii); (iv) antennae effects; (v) guest species that are fluorescent; (vi) excimer and exciplex emission; (vii) surface functionalization; and (viii) scintillation (Allendorf *et al.*, 2009 ▸). Regardless of the origin of the lumin­escence, it is well known that paramagnetic transition metal ions quench luminescence in general; the mechanism of quenching is supposedly by either an energy or a charge-transfer process based on *d*–*d* transitions (Allendorf *et al.*, 2009 ▸; Furman *et al.*, 2011 ▸; Jayaramulu *et al.*, 2012 ▸; Hu *et al.*, 2014 ▸). In a recent study, Ma and co-workers have shown that gradual postsynthetic metal node metathesis of a Cd-MOF with Mn^2+^ ions leads to a corresponding reduction in the fluorescence quantum yield of the Cd-MOF from 74.8 to 9.7% (Ma *et al.*, 2013 ▸). Although a number of LMOFs have been reported over the past decade or so, we are unaware of any LMOF based on a paramagnetic metal ion having been explored for sensing applications. Against this backdrop, we were motivated to explore the luminescence properties of an Mn-MOF constructed from a rationally designed pyrene-based fluorescent organic linker (see below). We chanced upon this Mn-MOF during our persistent, but unsuccessful, attempts aimed at accessing porous fluorescent MOFs from a brilliantly fluorescent pyrene-tetraacid linker with *d* ^10^ metal ions.

**Figure 1:**
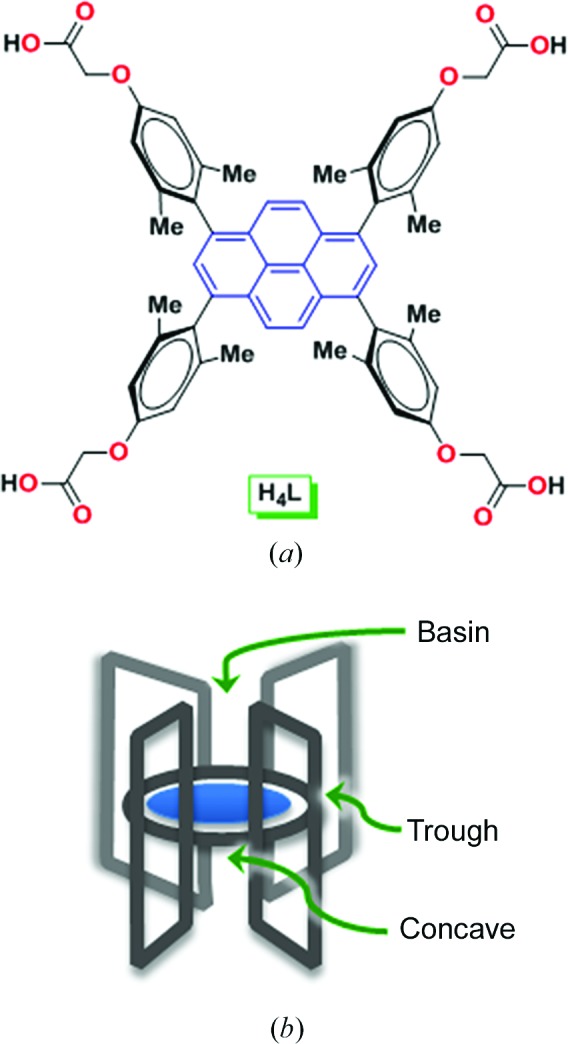
(*a*) The structure of the semi-rigid tetraacetic acid H_4_ *L*. (*b*) A representation of the molecular system with its three different domains, namely concave, trough and basin, for guest inclusion.

Our own interest has been centred on the development of organic and metal–organic porous materials based on a *de novo* design of molecular building blocks that inherently feature concave shapes (Moorthy & Natarajan, 2010 ▸; Moorthy *et al.*, 2010 ▸, 2011 ▸; Natarajan *et al.*, 2012 ▸; Bajpai *et al.*, 2012 ▸, 2013 ▸, 2014 ▸, 2015 ▸). In an extension of our studies of lattice inclusion chemistry, we sought to develop MOFs by metal-assisted self-assembly of a tetraacid, namely 1,3,6,8-tetra­kis[2,6-dimethyl-4-(α-carboxy)methoxyphenyl]pyrene (H_4_ *L*; Fig. 1 ▸). Our rationale for the design of this ligand involved several considerations, which were as follows. First, the linker H_4_ *L* is characterized by a planar pyrene core and orthogonally oriented flat aromatic rings, on which the methyl groups are strategically located to impart structural rigidity *via* steric considerations. Such a structure typifies a molecular system with three different domains, concave, trough and basin, for guest inclusion (Fig. 1 ▸). Indeed, analogous systems have been shown to lend themselves to the creation of multicomponent molecular crystals based on the binding of guests in different domains of the host in the solid state (Moorthy *et al.*, 2010 ▸; Bajpai *et al.*, 2012 ▸; Natarajan *et al.*, 2012 ▸). Second, the 4-connecting linker also features carboxy­methyl groups at the periphery of the aromatic rings. These groups were meant to impart some structural flexibility to the otherwise rigid organic linker, such that metal-assisted self-assembly might lead successfully to MOFs without any structurally imposed constraints. In this regard, flexible organic linkers have been known in the literature to afford metal–organic materials with one-dimensional infinite secondary building units (SBUs), thereby preventing the possibility of interpenetration (Rosi *et al.*, 2002*a* ▸,*b* ▸; Dai *et al.*, 2011 ▸; Zhan *et al.*, 2013 ▸). Third, guest binding in the three domains in close proximity to the pyrene fluorophore was surmised to signal guest location *via* changes in fluorescence. Specifically, our objectives were to access MOFs with *d* ^10^ metal ions, whereby the emission from the pyrene linker could be exploited for sensing applications. As mentioned above, extensive efforts to realise Cd- and Zn-MOFs with the tetraacid linker H_4_ *L* were unfruitful, while treatment with Mn(NO_3_)_2_ readily led to an Mn-MOF, Mn-*L*. Herein, we report that Mn-*L* exhibits appreciable lumin­escence to permit unprecedented exploitation of an MOF with paramagnetic metal nodes for the sensing of nitroaromatics. Further, we show that Mn-*L* uniquely displays a solvent-dependent emission maximum for application as a probe of solvent polarity.

## 2. Experimental   

### 2.1. General aspects   

^1^H NMR spectra were recorded with a JEOL Lambda (500 MHz) spectrometer. ^13^C NMR spectra were recorded with a 125 MHz NMR spectrometer with complete proton decoupling. IR spectra were recorded using a Bruker Vector 22 FT-IR spectrophotometer. Mass spectroscopic analyses were carried out with a Waters ESI-^Q^TOF instrument. The electron paramagnetic resonance (EPR) spectrum was recorded with a Bruker EMX EPR spectrometer. Powder X-ray diffractograms were recorded on a Rigaku MiniFlex600 X-ray diffractometer. Thermogravimetric analyses (N_2_ atmosphere, heating rate of 10 K min^−1^) were carried out with a Mettler–Toledo TGA apparatus. The melting points were determined with a JSGW melting-point apparatus. All the reactions were monitored by analytical thin-layer chromatography (TLC) using commercial aluminium sheets pre-coated with silica gel (Merck TLC silica gel 60F_254_). Column chroma­tography was conducted using silica gel of 100–200 µm mesh (Acme, Mumbai, India). All solvents were freshly distilled prior to use. Mn(NO_3_)_2_·6H_2_O and DMF were obtained from Sigma–Aldrich and used without any further purification.

### 2.2. Solvothermal synthesis of Mn-*L*   

To a solution of H_4_ *L* (50 mg, 0.055 mmol) in DMF–H_2_O (4:1 *v*/*v*, 12.5 ml) was added Mn(NO_3_)_2_·6H_2_O (36 mg, 0.125 mmol). The contents were dissolved by sonication, tightly capped in a glass vial and heated at 363 K. Crystals of Mn-*L* developed after 2 d (yield 84%, 58 mg, 0.046 mmol).

### 2.3. X-ray crystallography   

The X-ray diffraction intensity data collection for the crystals of Mn-*L* was carried out at 100 K on a Bruker Nonius SMART APEX CCD area-detector system with Siemens sealed ceramic Mo diffraction tube (λ = 0.7107 Å) and a highly oriented graphite monochromator, operating at 50 kV and 30 mA. The lattice parameters and standard deviations were obtained by a least-squares fit using 25 frames with 20 s frame^−1^ exposures with the Bruker *APEX2* software (Version 2012.10-0). Data were collected in a hemisphere mode by φ and ω scans, with 2θ = 40° and ∼10 s frame^−1^ exposures. Data processing and reduction were carried out using the Bruker *SAINT* software (Version 8.27B) and empirical absorption correction was done using the Bruker *SADABS* software (Version 2012/1). The structure was solved by direct methods using *WINGX* (Farrugia, 2012 ▸) and *SHELXL* (Sheldrick, 2008 ▸) and refined by the full matrix least-squares method based on *F* ^2^ using *SHELXLE2014* (Sheldrick, 2015 ▸). H atoms were treated as riding on their parent atoms and refined isotropically, while all non-H atoms were subjected to anisotropic refinement. All of the refinement process was performed before calculating the solvent-accessible void space using *Mercury* (Version 3.3; Macrae *et al.*, 2008 ▸). The X-ray crystallographic coordinates for the structure of Mn-*L* have been deposited at the Cambridge Crystallographic Data Centre (deposition No. CCDC 1057084).

### 2.4. Activation of Mn-*L*   

The as-synthesized crystalline compound (∼50 mg) was soaked in methanol. The supernatant methanol was discarded every 8 h (3–4 times) and fresh methanol was added each time. After methanol exchange, the sample was treated further in a similar way with acetone, and then with dichloro­methane to remove any remaining methanol and acetone. Finally, the supernatant dichloromethane was decanted and the MOF sample was dried under vacuum at 373 K for 12 h. The crystallinity of the resultant activated Mn-*L* was confirmed by powder X-ray diffraction (PXRD) analysis (see supporting information).

### 2.5. Steady-state fluorescence spectroscopy   

All the steady-state fluorescence measurements were performed at 298 K on a FluoroMax-4 FM4-3000 spectrofluorometer (Horiba Jobin Yvon Technology), which was standardized with an R928 photomultiplier tube, a DM302 photon-counting acquisition module biased at 950 V, a 1200 lines mm^−1^ grating blazed at 330 nm in the excitation monochromator and a 1200 lines mm^−1^ grating blazed at 500 nm in the emission monochromator. The slit widths for both excitation and emission were fixed at 2.00 nm bandpass and the accuracy in measuring the wavelength was ±2 nm. The solid-state fluorescence quantum yield determinations were performed using a QUANTA-φ F-3029 Horiba Scientific integrating sphere (sphere inner diameter: 150 mm) connected to the spectrofluorometer.

### 2.6. Solvatochromic studies   

The solvatochromic studies of Mn-*L* were performed with high-performance liquid chromatography (HPLC) grade solvents obtained from Fischer Scientific, India. In a typical process, the crystals of activated Mn-*L* (∼20 mg) were immersed in various solvents (5 ml) for 2 d. Subsequently, the solvent was decanted in each case and the crystals of Mn-*L* were air dried. The resultant solvent-included crystals were then employed for recording the fluorescence emission spectra.

### 2.7. Fluorescence quenching titration experiments   

Fluorescence quenching studies were performed using a dispersion of Mn-*L* in dichloromethane at 298 K. Typically, the dispersion was prepared by adding crystals of Mn-*L* (1 mg) to dichloromethane (3 ml), followed by sonication for 30 min. The resultant dispersion was subsequently placed in a quartz cell of 1 cm width. Finally, all the fluorescence titrations were carried out with excitation at 320 nm, by gradually adding solutions of various nitroaromatic analytes (in dichloro­methane) to the dispersion of Mn-*L* in dichloromethane. Each titration was repeated at least three times to obtain concordant values.

## 3. Results and discussion   

### 3.1. Synthesis of the semi-rigid tetraacetic acid H_4_ *L*   

**Figure 2:**
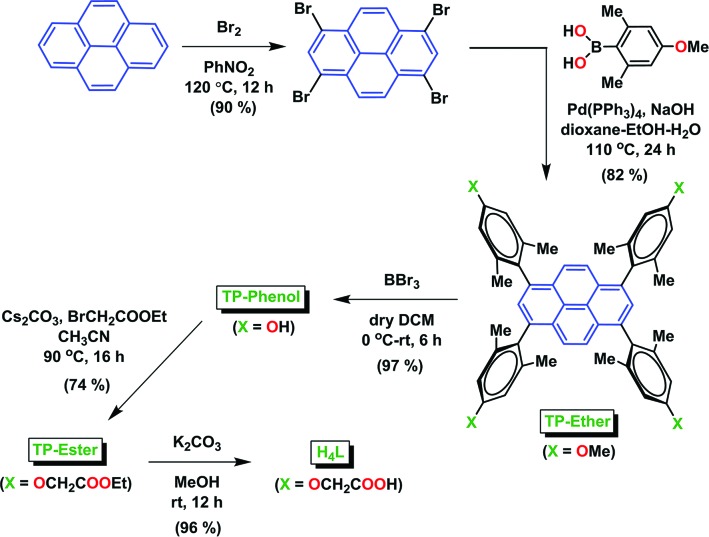
The synthesis of the semi-rigid tetraacetic acid H_4_ *L*.

The target tetratopic linker H_4_ *L* was synthesized according the route shown in Fig. 2 ▸. Thus, bromination of pyrene with Br_2_ in nitromethane at 393 K yielded 1,3,6,8-tetra­bromo­pyrene in a quantitative yield (Mikroyannidis, 2005 ▸). This was subjected to Suzuki coupling with 2,6-dimethyl-4-methoxy­phenylboronic acid under Pd(0)-catalysed conditions in dioxane–ethanol–water (4:2:1 *v*/*v*) at 383 K. The resultant tetraanisyl derivative (TP-Ether) was demethylated with BBr_3_ to afford the tetraphenol (TP-Phenol) in 97% yield. This was subjected to alkylation with ethyl α-bromoacetate in aceto­nitrile using Cs_2_CO_3_ as a base to yield the tetraester (TP-Ester), hydrolysis of which in K_2_CO_3_–MeOH furnished the required tetraacetic acid H_4_ *L* in a near-quantitative yield.

### 3.2. Synthesis and X-ray crystal structure determinations of Mn-MOF with tetraacetic acid linker H_4_ *L*   

**Figure 3:**
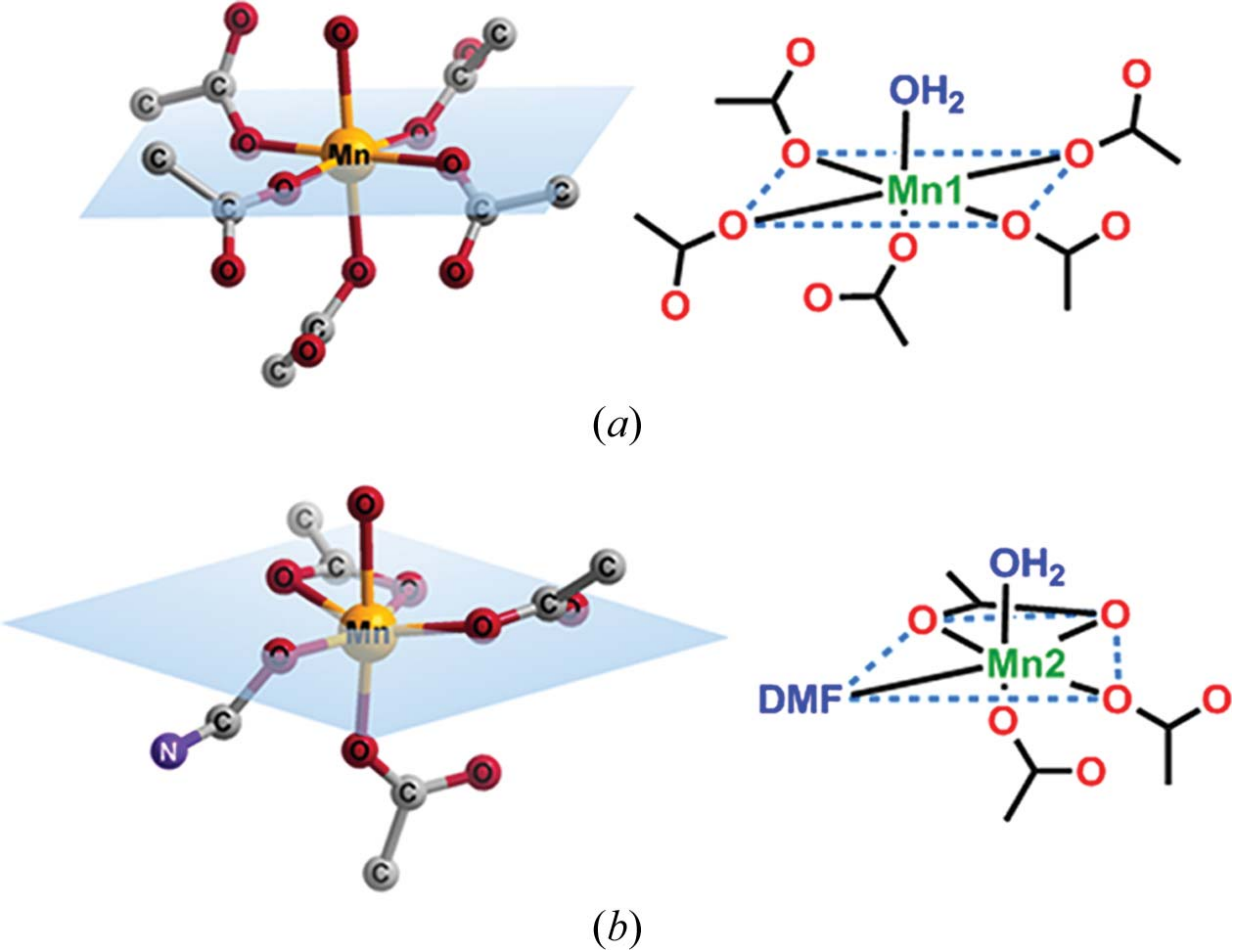
The coordination environments of (*a*) Mn1 and (*b*) Mn2 in Mn-*L*.

**Figure 4:**
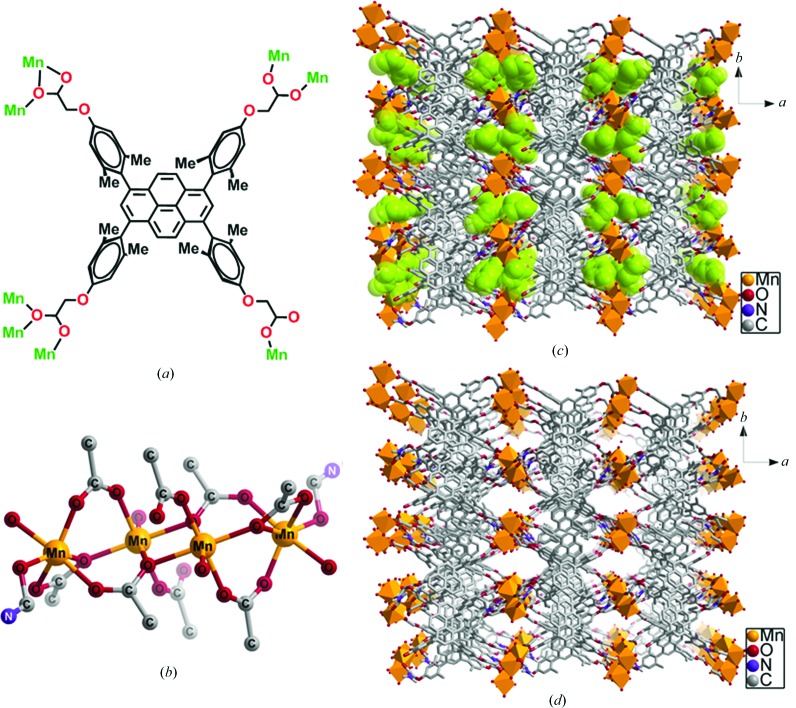
(*a*) The coordination modes of the carboxylate groups in *L*. (*b*) The tetrametallic SBU comprising Mn1 and Mn2. (*c*) A crystal packing diagram with and (*d*) without DMF guests (green).

Treatment of a solution of H_4_ *L* with Mn(NO_3_)_2_·6H_2_O in DMF–H_2_O (4:1 *v*/*v*) at 383 K in a tightly capped glass vial led to cube-shaped crystals of Mn-*L* after 48 h. X-ray single-crystal structure determination revealed that the crystals belong to the monoclinic system in space group *P*2_1_/*c* (Table 1 ▸). The asymmetric unit cell contains one *L* tetra-anion, two Mn^2+^ cations, one water molecule and three DMF molecules. Thus, the molecular formula of the repeat unit is [Mn_2_(*L*)(DMF)(H_2_O)]·2DMF. The crystal structure analysis also revealed that there are two crystallographically independent Mn^2+^ cations, Mn1 and Mn2, which have a distorted octahedral geometry (Fig. 3 ▸). In fact, Mn1 is coordinated by five O atoms from five carboxylate groups and by one water molecule, while Mn2 is coordinated by four O atoms of three carboxylate groups, and by one water molecule and one DMF molecule (Fig. 3 ▸). The coordination modes of the carboxylate groups in tetracarboxylate *L* are depicted in Fig. 4 ▸. The two Mn centres, Mn1 and Mn2, are bridged by three carboxylate groups, as shown in Fig. 4 ▸. The space-group symmetry of the crystalline MOF leads to a tetranuclear cluster in which the centrosymmetrically related Mn1 ions are connected by a μ_2_-COO bridge. As a result, four Mn^2+^ cations make up an 8-connecting SBU (Fig. 4 ▸). This, along with the 4-connecting organic spacer *L*, leads to a porous open framework structure with channels that propagate down the *c* axis (Fig. 4 ▸). Thus, the crystals represent a porous MOF, Mn-*L*, with the channels occupied by DMF molecules. The solvent-accessible volume with the exclusion of all DMF and H_2_O molecules was calculated to be 25.5% using *Mercury* (grid step = 0.2 Å and probe radius = 1.2 Å).

The synthesis of the crystals of the Mn-MOF, Mn-*L*, was readily adapted to a bulk scale, as established from the similarity between the PXRD profiles of pristine Mn-*L* and the simulated profile based on the structure determined by single-crystal X-ray crystallography (supporting information). The thermal stability of the Mn-MOF was examined by thermogravimetric (TGA) analysis. The TGA profile of Mn-*L* reveals a solvent loss corresponding to 17% up to ∼573 K (supporting information). The resulting MOF was stable up to 723 K, followed by thermal decomposition. Importantly, the crystals of Mn-*L* remained highly crystalline even after desolvation by heating the crystal at 373 K under vacuum for 12 h, as revealed by the PXRD analysis (supporting information).

### 3.3. Solvent-dependent photoluminescence of Mn-*L*   

**Figure 5:**
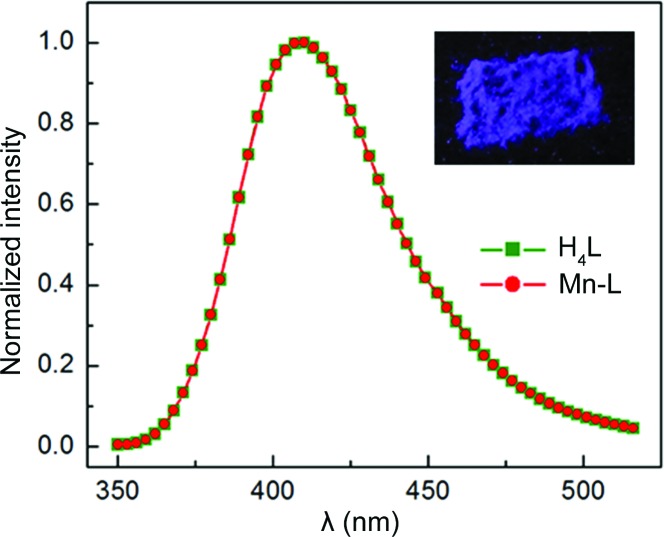
Fluorescence emission spectra of H_4_ *L* (green line) and Mn-*L* (red line) for excitation at 320 nm in the solid state. Note that the emission spectra of H_4_ *L* and Mn-*L* are identical. In the inset is shown a solid-state fluorescence image of the crystals of Mn-*L*.

As far as the photophysical properties of Mn-*L* are concerned, a moderate emission in the solid state was observed upon exposure of the material to UV light. Fig. 5 ▸ shows the solid-state emission spectrum of Mn-*L*, with an emission maximum at 410 nm for excitation at 320 nm; the broad emission is independent of excitation wavelength in the region between 250–350 nm. Ironically, the solid-state fluo­res­cence emission spectrum of the precursor organic linker H_4_ *L* is identical to that of Mn-*L*. This is not surprising given that the orthogonally oriented aryl rings at the four corners of the fluorescent pyrene insulate the latter. For both Mn-*L* and H_4_ *L*, solid-state fluorescence quantum yields were determined with an integrating sphere setup. The emission quantum yields for Mn-*L* and H_4_ *L* were determined to be 8.3 and 26%, respectively, for excitation at 320 nm. An observed emission quantum yield of 8.3% is indeed surprising, as paramagnetic metal ions are known to quench the emission, as mentioned earlier.

**Figure 6:**
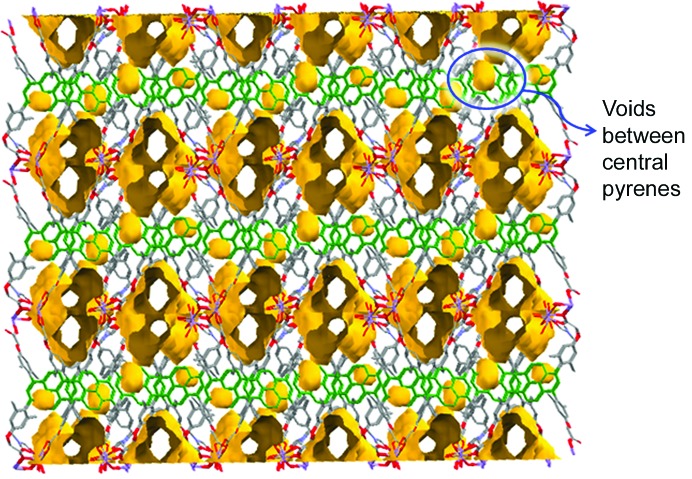
Void volumes in the crystal structure of Mn-*L*, as revealed by *Mercury*, with a grid step of 0.2 Å and probe radius of 1.2 Å. Note that the voids are largely located near the concave region of the tetraarylpyrene scaffold.

This respectable emission and the significant solvent-accessible volume of ∼25.5%, as revealed from the three-dimensional porous framework of Mn-*L*, encouraged us to explore whether the latter could be applied as a material to signal guest binding. The crystal structure analyses (using *Mercury*) show that the void spaces in the crystal lattice of Mn-*L* exist largely in the proximity of the pyrene core. In this regard, it is amply evident that there is very scant void volume in the so-called basin region of the pyrene core, while significant void volumes are located in the concave region of the tetraarylpyrene scaffold (Fig. 6 ▸). We thus envisioned that changes in the fluorescence properties of the MOF in the presence of guests should relay information about the latter.

**Figure 7:**
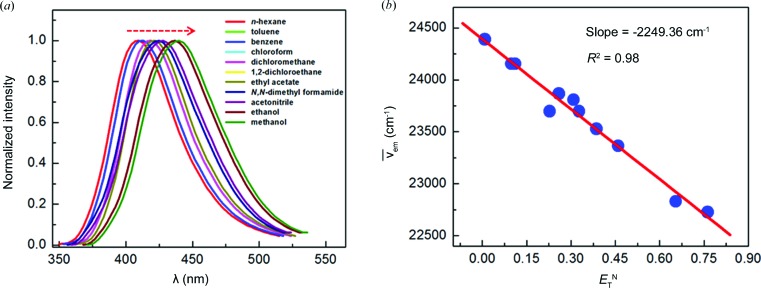
(*a*) Solid-state fluorescence emission spectra of Mn-*L* after various solvent inclusions (λ_ex_ = 320 nm). (*b*) The linear correlation between the emission maximum of solvent-included Mn-*L* and Reichardt’s solvent polarity parameter (*E*_T_^N^).

To begin with, we wondered if the Mn-*L* MOF exhibits solvent-dependent emission when exposed to different solvents. Thus, solvent-dependent emission studies were carried out as follows. Crystals of pristine Mn-*L* were activated and immersed in a given solvent for 2 d. Subsequently, the crystals were air-dried and fluorescence spectra were recorded. The stabilities of such MOF crystals were examined by PXRD, which revealed that their structural integrity was conserved during this process. This procedure was followed uniformly for fluorescence measurements of Mn-*L* MOF in 11 different solvents of varying polarities, as shown in Table 2 ▸. Notably, the emission maximum of Mn-*L* was found to vary over a range of 30 nm depending on the solvent polarity, undergoing a progressive red shift from 410 nm in a nonpolar solvent such as *n*-hexane to 440 nm in a polar solvent such as methanol (Fig. 7*a* ▸). Clearly, the Mn-*L* MOF acts as a solvent polarity probe. Fig. 7(*b*) ▸ shows the correlation between the observed emission maxima and Reichardt’s solvent polarity parameter *E*_T_^N^ (Reichardt, 1994 ▸). One observes a linear regression with excellent goodness of fit (*R* ^2^ = 0.98). Although MOFs have been demonstrated as solvatochromic materials (Lu *et al.*, 2011 ▸; Mehlana *et al.*, 2012 ▸; Mehlana, Bourne, Ramon & Öhrström, 2013 ▸; Mehlana, Ramon & Bourne, 2013 ▸; Cui *et al.*, 2013 ▸; Mallick *et al.*, 2015 ▸), an appreciable correlation of the emission maxima with solvent polarity parameters is heretofore unknown.

### 3.4. Luminescent Mn-*L* as applied to sensing of nitroaromatic explosives   

**Figure 8:**
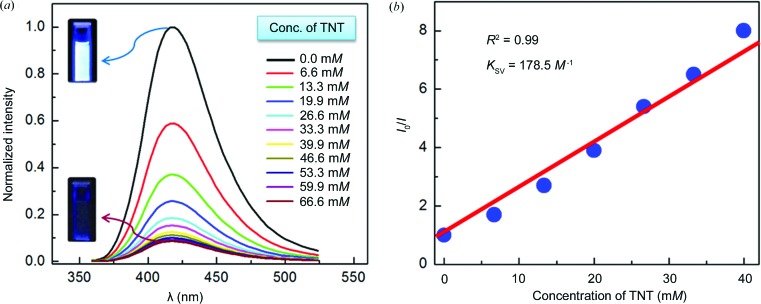
(*a*) Quenching of the fluorescence intensity of Mn-*L* with increasing concentration of TNT in dichloromethane (λ_ex_ = 320 nm). Note the changes in the fluorescence images (insets) of the dispersed MOF before and after quenching. (*b*) Stern–Volmer plot with increasing concentration of TNT.

We were tempted by the presence of appreciable void volume in the crystals of Mn-*L* and the respectable fluor­escence quantum yields of crystalline Mn-*L* to explore guest binding using fluorescence. It was surmised that the electron-rich aromatic pyrene linker (*L*) should relay information about its interaction with any electron-deficient (π-acceptor) aromatic guest species through changes in fluorescence. We thus chose to examine the binding/sensing of trace quantities of hazardous and explosive nitroaromatic compounds, namely nitrobenzene (NB), 4-nitrotoluene (NT), 1,3-dinitrobenzene (DNB), 2,4-dinitrotoluene (DNT) and 2,4,6-trinitrotoluene (TNT). Accordingly, fluorescence quenching titrations were carried out with the incremental addition of each of the nitroaromatic analytes to crystals of Mn-*L* dispersed in dichloromethane; the structural integrity of the Mn-*L* crystals upon dispersion in dichloromethane with and without the added nitroaromatic was confirmed to be intact by PXRD analysis. With increasing concentration of each of the nitroaromatics, a gradual decrease in the fluorescence intensity of the suspension of Mn-*L* in dichloromethane was observed (supporting information). A representative fluorescence quenching titration of Mn-*L* with TNT is shown in Fig. 8 ▸. The steady-state fluorescence quenching data could be readily subjected to a linear regression analysis following the Stern–Volmer equation
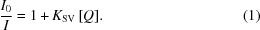
Remarkably, the sensing of the nitroaromatic compounds by Mn-*L* by fluorescence quenching can be readily made out with the naked eye (Fig. 8 ▸).

**Figure 9:**
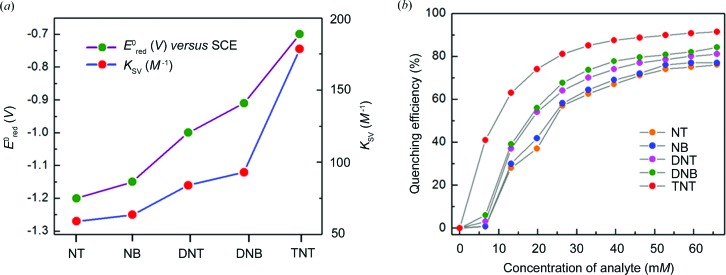
(*a*) The correlation between reduction potentials and Stern–Volmer quenching constants of various nitroaromatic analytes. (*b*) A plot of quenching efficiency *versus* concentration for each of the nitroaromatic analytes.

The Stern–Volmer quenching constants (*K*_SV_s) thus derived for all the nitroaromatic analytes follow the order: NT (59.0 *M* ^−1^) < NB (63.5 *M* ^−1^) < DNT (84.0 *M* ^−1^) < DNB (93.0 *M* ^−1^) < TNT (178.5 *M* ^−1^) (Table 3 ▸). This result indeed reveals the fact that the fluorescence quenching process becomes much faster and driven thermodynamically more and more favourably with increasingly electron-deficient nitro­aromatics. The fact that fluorescence quenching becomes progressively more facile for electron-deficient analytes (such as nitroaromatics) only when their lowest unoccupied mol­ecular orbitals (LUMOs) are located between the valence band (VB) and conduction band (CB) of an electron-rich MOF is well documented in the literature (Nagarkar *et al.*, 2013 ▸; Pramanik *et al.*, 2013 ▸; Gole *et al.*, 2014 ▸). Thus, upon photoexcitation, electron transfer may occur from the CB of the MOF to the LUMO of the electron-deficient analyte, leading to a gradual diminution in the fluorescence intensity of the MOF. The Stern–Volmer rate constants determined for the fluorescence quenching of the Mn-*L* MOF by various aromatics correlate very well with the reduction potentials of the latter (Peover, 1964 ▸; Gole *et al.*, 2014 ▸) (Table 3 ▸ and Fig. 9 ▸). As can be seen, the highest quenching constant is observed for TNT, with the lowest LUMO energy and highest reduction potential, while the lowest quenching constant is found for the case of NT, with the lowest reduction potential.

We also wished to assess the fluorescence quenching efficiency by various nitroaromatics in increasing concentrations. In Fig. 9 ▸ are shown the plots of quenching efficiency *versus* nitroaromatic concentration; the quenching efficiency (η) is defined by the relation
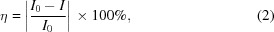
(Lan *et al.*, 2009 ▸). It thus emerges clearly that the fluorescence quenching efficiency increases gradually with increasing concentration, and that it is highest for the most electron-deficient aromatic compound, namely TNT. In fact, ∼74% quenching of fluorescence occurs at ∼20 m*M* concentration of TNT, while only 37% quenching occurs for NT at the same concentration (Table 3 ▸).

To estimate the sensitivity limits of the Mn-*L* MOF for detection of TNT, quenching experiments were performed at low concentration levels (∼10^−4^ *M*). A plot of the quenching efficiency (%) *versus* the quencher concentration in this range reveals that the minimum (limiting) concentration for the detection of TNT is 5.5 × 10^−4^ *M* (supporting information). One may thus calculate that the Mn-*L* MOF allows the detection of TNT at a level of 125 p.p.m. Furthermore, quenching and recovery experiments were carried out to investigate the stability and recyclability of the Mn-*L* MOF for the detection of TNT. Interestingly, the original fluorescence intensity of Mn-*L* could easily be restored by centrifugation of the dispersed MOF solution (after quenching titration) in dichloromethane, followed by washing three or four times with dichloromethane and ethanol, and drying under vacuum. The fluorescence intensity of Mn-*L* remained almost the same even after five cycles, which attests to the excellent photostability of the Mn-*L* MOF (supporting information).

### 3.5. Insights from the fluorescent Mn-*L* MOF and implications for guest signalling   

One of the serious limitations with the synthesis of MOFs is that one cannot access with certainty the theoretically plausible porous crystals of predefined topology for a given organic linker and a metal ion; a variety of factors, including experimental conditions, the nature of the counter-anion and the energetics associated with the formation of the framework structure, play a decisive role. Therefore, to access MOFs with a given organic linker, one must experiment with different conditions as well as with different metal ions. While some metal ions with a particular connectivity lead to MOFs, some other metal ions with the same geometric connectivities do not necessarily lead to MOFs. Indeed, this is the case with the organic linker H_4_ *L*, which led to the Mn-*L* MOF, while our best efforts to access MOFs with *d* ^10^ metal ions were in vain. Notwithstanding the notion that paramagnetic metal ions quench fluorescence, we believed that fixation of the metal ions in MOFs into certain geometries through organic linkers might prohibit the mechanisms of quenching. It was thus gratifying to observe respectable fluorescence from the Mn-*L* MOF, which attests to the fact that MOFs based on fluorescent linkers and paramagnetic metal ions might indeed display notable fluorescence to permit their utility for fluorescence-based sensing applications. One of the reasons why the Mn-*L* MOF exhibits respectable quantum yields of fluorescence can be traced to the structural attributes of the ligand H_4_ *L*, in which the pyrene fluorophore is protected by the orthogonally installed dimethylaryl rings; the latter may at least attenuate the efficiency of fluorescence quenching, if not completely prohibit it. The observed quantum yield of fluorescence of ∼8.3% for the Mn-*L* MOF is significantly lower than that of the free tetraacid linker H_4_ *L* (26%). The rigidification of the Mn-*L* MOF with the inclusion of metal ions should manifest in an enhancement of the quantum yield, which is contrary to what is observed. We believe that the metal ions that are part of the framework structure may interact with the fluorophores of neighbouring linkers to allow the quenching mechanism to become effective to a certain degree. Nonetheless, it emerges from our observations of the Mn-*L* MOF that porous MOFs constructed from paramagnetic metal ions and organic linkers with protected fluorophores could still be fluorescent.

Although MOFs have been found to exhibit remarkable solvent-dependent changes in their emission properties, we are unaware of a correlation between solvent polarity parameters and the observed changes in the emission maximum. As shown in Fig. 7 ▸, the Mn-*L* MOF lends itself to an excellent correlation between the emission maxima in different solvents and their *E*_T_^N^ polarity parameters. Furthermore, the fact that such a MOF has application in sensing is demonstrated by quenching of the fluorescence with electron-deficient nitroaromatics. Indeed, one observes a remarkable correlation between the Stern–Volmer quenching constants and the reduction potentials of the nitroaromatic quenchers. The limit for detection of TNT is 125 p.p.m. Although much better limits have been documented for fluorescent MOFs (Gole *et al.*, 2014 ▸), we consider the results invaluable and truly remarkable given that the fluorescent MOF is based on a paramagnetic metal ion.

An incisive structural analysis may shed light on the locations of the binding of nitroaromatic compounds and offer insights as to the detection limits. Fig. 6 ▸ depicts the void volumes in crystals of the Mn-*L* MOF as revealed by the *Mercury* program. It is amply evident that there is a very scant void volume in the so-called basin region of the pyrene core, while significant void spaces are located in the concave regions of the linker *L*. Thus, the binding of guest species in this region cannot be expected to be the best for quenching. These analyses thus point to the fact that MOFs based on linkers with modified structural attributes that allow the creation of voids in the proximity of the basin region of the fluorophore may permit the detection of TNT with much higher sensitivity.

While fluorescent MOFs are being increasingly explored for applications such as chiral sensing (Wanderley *et al.*, 2012 ▸; Hu *et al.*, 2014 ▸), biosensing (Wu, Gong *et al.*, 2012 ▸; Wu, Wang *et al.*, 2012 ▸; Xu *et al.*, 2012 ▸; Wei *et al.*, 2013 ▸; Hu *et al.*, 2014 ▸), sensing of explosives (Lan *et al.*, 2009 ▸; Xu *et al.*, 2011 ▸; Nagarkar *et al.*, 2013 ▸; Pramanik *et al.*, 2013 ▸; Gole *et al.*, 2014 ▸; Hu *et al.*, 2014 ▸; Zhang *et al.*, 2014 ▸; Liu *et al.*, 2015 ▸; Zhang *et al.*, 2015 ▸), detection of volatile organic compounds (Hu *et al.*, 2014 ▸; Zhang *et al.*, 2014 ▸; Zhan *et al.*, 2014 ▸) and ionic species (He *et al.*, 2013 ▸; Tang *et al.*, 2013 ▸; Hu *et al.*, 2014 ▸), luminescent thermometers (D’Vries *et al.*, 2013 ▸; Rao *et al.*, 2013 ▸; Shustova *et al.*, 2013 ▸; Hu *et al.*, 2014 ▸) *etc.*, we are excited about the opportunities provided by fluorescent MOFs based on paramagnetic metal ions for the detection of paramagnetic molecular free radicals. The latter are invaluable chemical intermediates that exist in different environments, such as those in combustion (Cramer & Campbell, 1949 ▸; Valavanidis *et al.*, 2008 ▸; Davis & Francisco, 2014 ▸), in the atmosphere (Heard & Pilling, 2003 ▸; Crounse *et al.*, 2013 ▸) and in biological systems (Valko *et al.*, 2007 ▸; Alamed *et al.*, 2009 ▸; Hong *et al.*, 2010 ▸; Panasenko *et al.*, 2013 ▸; Song *et al.*, 2014 ▸).

## 4. Conclusions   

A rationally designed organic tetraacid linker H_4_ *L* with inherent concave features was exploited for the synthesis of porous MOFs. While our efforts to access MOFs with *d* ^10^ metal ions such as zinc(II) and cadmium(II) were unsuccessful, treatment of H_4_ *L* with Mn(NO_3_)_2_ led readily to an Mn-MOF, Mn-*L*, with 25% solvent-accessible volume. Notwithstanding the notion that paramagnetic metal ions quench fluorescence, our investigations revealed that the crystals of Mn-*L* exhibit appreciable fluorescence; indeed, the solid-state fluorescence quantum yield was determined to be 8.3%. Remarkably, the Mn-*L* MOF exhibits a solvent-dependent emission maximum such that the emission maxima in different solvents give rise to an excellent and unprecedented correlation with Reichardt’s solvent polarity parameter (*E*_T_^N^). It has been shown that Mn-*L* can find application in the detection of nitroaromatic compounds, with the detection limit for 2,4,6-trinitrotoluene (TNT) being 125 p.p.m. An incisive analysis of the crystal structure offers insights concerning the appreciable fluorescence observed for the Mn-*L* MOF, and the factors that might increase the detection limits of nitroaromatics.

## 5. Supporting information   

Full details of the synthesis of the starting materials and their spectroscopic data, ^1^H and ^13^C NMR spectra of all the compounds, IR spectrum, EPR spectrum, TGA profile and solid-state excitation spectra of Mn-*L*, details of PXRD analyses, details of the fluorescence quenching titrations and determination of the Stern–Volmer quenching constants for NT, NB, DNT and DNB, together with a full CIF and structure factors, are available in the supporting information.

## Supplementary Material

Crystal structure: contains datablock(s) Mn-L. DOI: 10.1107/S2052252515012506/ed5005sup1.cif

Structure factors: contains datablock(s) Mn-L. DOI: 10.1107/S2052252515012506/ed5005Mn-Lsup2.hkl

Supporting information. DOI: 10.1107/S2052252515012506/ed5005sup3.pdf

CCDC reference: 1057084

## Figures and Tables

**Table 1:** Crystal data and refinement parameters for Mn-*L*

Parameters	Mn-*L*
Empirical formula	C_65_H_67_O_17_N_3_Mn_2_
Formula weight	1272.10
Temperature (K)	100(2)
Wavelength ()	0.71073
Crystal habit	Cube
Crystal colour	Colourless
Crystal system	Monoclinic
Space group	*P*2_1_/*c* (No. 14)
*a* ()	19.6786(11)
*b* ()	22.7703(13)
*c* ()	14.8344(9)
()	90
()	104.15(1)
()	90
Volume (^3^)	6446(1)
*Z*	4
Calculated density (Mgm^3^)	1.311
Absorption coefficient (mm^1^)	0.462
*F*(000)	2660
range for data collection ()	2.0828.34
Index ranges	26 *h* 26
	26 *k* 30
	19 *l* 19
Reflections collected	54349
Refinement method	Full-matrix least-squares on *F* ^2^
Data, restraints, parameters	16040, 0, 726
Goodness-of-fit on *F* ^2^	1.047
Final *R* indices [*I* > 2(*I*)]	*R*_1_ = 0.1062, *wR*_2_ = 0.2780
*R* indices (all data)	*R*_1_ = 0.1754, *wR*_2_ = 0.3136
Solvent-accessible volume (^3^)	1643.73

**Table 2:** Solvatochromic absorption data for solvent-included Mn-*L* crystals

Solvent	*E*_T_^N^	_max_^em^ (nm) of solvent-included Mn-*L* crystals	Wavenumber (cm^1^)
*n*-Hexane	0.009	410	24390.2
Toluene	0.099	414	24154.6
Benzene	0.111	414	24154.6
Chloroform	0.259	419	23866.4
Dichloromethane	0.309	420	23809.5
1,2-Dichloroethane	0.327	422	23696.6
Ethyl acetate	0.228	422	23696.6
*N*,*N*-Dimethyl formamide	0.386	425	23529.4
Acetonitrile	0.46	428	23364.5
Ethanol	0.654	438	22831.1
Methanol	0.762	440	22727.3

**Table 3:** Reduction potentials and fluorescence quenching data for various nitroaromatics

Nitroaromatic analyte	Reduction potential (V *versus* saturated calomel electrode)	*K*_sv_ (*M* ^1^)	Quenching efficiency (%) at 20m*M* concentration of analyte
NT	1.20	59.0	37
NB	1.15	63.5	42
DNT	1.00	84.0	54
DNB	0.91	93.0	56
TNT	0.70	178.5	74
