# Supplementary material for: A fluorescent paramagnetic Mn metal–organic framework based on semi-rigid pyrene tetra­carboxylic acid: sensing of solvent polarity and explosive nitroaromatics
Source: IUCrJ. 2015 Aug 14;2(Pt 5):552–62. doi: 10.1107/S2052252515012506 (PMC4547823; doi:10.1107/S2052252515012506)
Supplement: Supplementary file 3 [file m-02-00552-sup3.pdf]

SUPPORTING INFORMATION

A Fluorescent Paramagnetic Mn-MOF Based on Semi-Rigid Pyrene-Tetracarboxylic Acid: Sensing of Solvent Polarity and Explosive Nitroaromatics

Alankriti Bajpai,[§] Arindam Mukhopadhyay,[§] Manchugondanahalli Shivakumar Krishna,[§] Savitha Govardhan,[§] and Jarugu Narasimha Moorthy[§,*]

[§] *Department of Chemistry, Indian Institute of Technology, Kanpur-208016, INDIA*

E-mail: moorthy@iitk.ac.in

CONTENTS

1.	Synthesis of $\mathbf{H_4L}$	S2
3.	IR spectrum of **Mn-L**	S6
4.	Electron Paramagnetic Resonance (EPR) spectrum of **Mn-L**	S6
5.	TGA profile of **Mn-L**	S7
6.	Solid-state excitation spectrum of **Mn-L**	S7
7.	PXRD patterns of variuos solvent-included **Mn-L** crystals	S8
8.	PXRD patterns of **Mn-L** crystals obtained after immersing in solution of various nitroarmatic analytes in dichloromethane	S8
9.	Fluorescence quenching titrations of **Mn-L** with NT	S9
10.	Fluorescence quenching titrations of **Mn-L** with NB	S9
11.	Fluorescence quenching titrations of **Mn-L** with DNT	S10
12.	Fluorescence quenching titrations of **Mn-L** with DNB	S10
13.	Determination of the sensitivity of **Mn-L** towards the detection of TNT	S11
14.	Quenching and recovery test of **Mn-L** with TNT	S11
15.	^{1}H (500 MHz, $CDCl_3$) and ^{13}C NMR (125 MHz, $CDCl_3$) spectra of 1,3,6,8-tetrakis(2,6-dimethyl-4-methoxyphenyl)pyrene	S12
16.	^{1}H (500 MHz, DMSO-d_6) and ^{13}C NMR (125 MHz, DMSO-d_6) spectra of 1,3,6,8-tetrakis(2,6-dimethyl-4-hydroxyphenyl)pyrene	S13
17.	^{1}H (400 MHz, $CDCl_3$) and ^{13}C NMR (100 MHz, $CDCl_3$) spectra of 1,3,6,8-tetrakis(2,6-dimethyl-4-(α-carboethoxy)methoxyphenyl)pyrene	S14
18.	^{1}H (500 MHz, DMSO-d_6) and ^{13}C NMR (125 MHz, DMSO-d_6) spectra of 1,3,6,8-tetrakis(2,6-dimethyl-4-(α-carboxy)methoxyphenyl)pyrene, $\mathbf{H_4L}$.	S15
19.	Checkcif report+explanation	S16

Synthesis of H_4L.

1,3,6,8-Tetrakis(2,6-dimethyl-4-methoxyphenyl)pyrene: TP-Ether

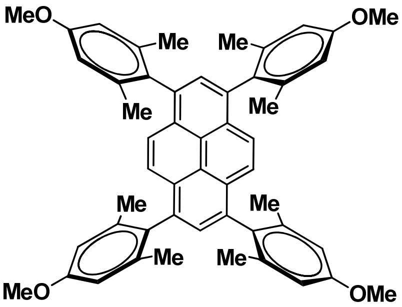

An oven-dried pressure tube of 100 mL volume was cooled under a N_2 gas atmosphere and charged with 1,3,6,8-tetrabromopyrene (1.0 g, 1.93 mmol), 2,6-dimethyl-4-methoxyphenylboronic acid (2.78 g, 15.4 mmol), $Pd(PPh_3)_4$ (0.56 g, 0.48 mmol), powdered NaOH (0.927 g, 23.16 mmol), 30 mL of dioxane, 20 mL of EtOH and 2 mL of distilled water. The resultant mixture was purged with a N_2 gas for 15 min, the pressure tube was capped tightly and heated at 110 °C. The yellow turbid solution turned to a clear one within 30 min. The heating was continued for 24 h, after which time the color of the reaction mixture turned dark brown. It was then cooled, solvent mixture removed under vacuo and the solid residue was extracted with DCM. The combined DCM extract was washed with brine solution, dried over anhyd Na_2SO_4 and concentrated. The pure product was isolated (as a colorless solid) by silica gel column chromatography using $CHCl_3$/pet. ether (50:50, v/v) mixture as an eluent; yield 82% (1.16 g, 1.57 mmol); mp 248–252 °C; IR (KBr) cm^{-1} 2922, 2852, 1598; ^1H NMR (400 MHz, $CDCl_3$) δ 1.95 (s, 24H), 3.86 (s, 12H), 6.75 (s, 8H), 7.51 (s, 4H), 7.61 (s, 2H); ^{13}C NMR (100 MHz, $CDCl_3$) δ 21.1, 55.3, 112.7, 124.8, 125.9, 128.8, 129.7, 132.7, 136.0, 138.3, 158.7; ESI-MS *m/z* calcd for $C_{52}H_{51}O_4$ 739.3787, found 739.3743 $(M+H)^+$.

1,3,6,8-Tetrakis(2,6-dimethyl-4-hydroxyphenyl)pyrene: TP-Phenol

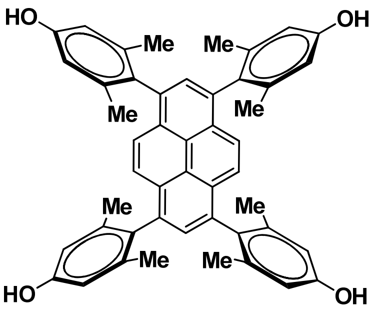

To a solution of methyl aryl ether **1** (1.0 g, 1.35 mmol) in 30 mL of dry DCM at 0 °C was added BBr_3 (1.62 g, 6.49 mmol) dropwise. The reaction mixture was allowed to stir for 6 h at rt. Subsequently, it was quenched with ice-cold water, extracted with ethyl acetate, washed with brine solution, dried over anhyd Na_2SO_4, filtered and concentrated. The pure product was obtained as a colorless solid after filtration over a short pad of silica gel using a mixture of ethyl acetate/pet. ether (60:40, v/v); yield 97% (0.924 g, 1.35 mmol); mp > 300 ºC; IR (KBr) cm^{-1} 3320, 3050, 2925, 1591, 1476, 1429; ^1H NMR (500 MHz, DMSO-d_6) δ 1.81 (s, 24H), 6.64 (s, 8H), 7.45 (s, 2H), 7.47 (s, 4H); ^{13}C NMR (125 MHz, DMSO-d_6) δ 21.0, 114.8, 124.9, 125.8, 128.6, 128.8, 130.56, 136.6, 137.7, 157.0; ESI-MS *m*/*z* calcd for $C_{48}H_{43}O_4$ 683.3004, found 681.3117 $(M–H)^-$.

1,3,6,8-Tetrakis(2,6-dimethyl-4-(α-carboethoxy)methoxyphenyl)pyrene: TP-Ester

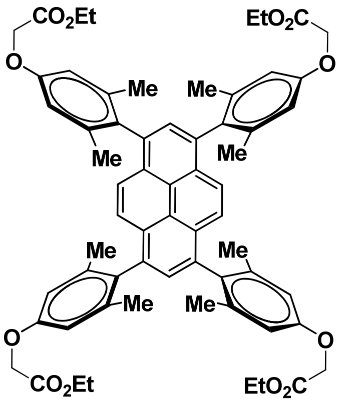

In a two-necked round bottom flask equipped with a $CaCl_2$ guard tube were taken tetraphenol **2** (1.0 g, 2.33 mmol), Cs_2CO_3 (7.6 g, 23.39 mmol) and 40 mL of anhyd CH_3CN. After stirring the contents for 10 min, ethyl α-bromoacetate (4.68 g, 28.07 mmol) was introduced. Subsequently, the reaction mixture was heated at reflux overnight. After 16 h, the reaction was judged to be complete by TLC analysis. The reaction was quenched by addition of 10 mL of distilled H_2O. Acetonitrile was removed under vacuo and the solid residue was extracted with DCM and washed with brine solution. The combined organic phase was dried over anhyd Na_2SO_4 and concentrated in vacuo. The excess ethyl bromoacetate was removed by applying high-vacuum. Silica gel column chromatography of the crude reaction mixture using a mixture of ethyl acetate/pet. ether (30:70, v/v) as an eluent yielded the pure product as a colorless solid; yield 74% (1.11 g, 1.08 mmol); mp 248–252 °C; IR (KBr) cm^{-1} 2976, 2922, 2868, 1757, 1607, 1583, 1476, 1446; ^{1}H NMR (400 MHz, $CDCl_3$) δ 1.32 (t, J = 6.9 Hz, 12H), 1.93 (s, 24H), 4.31 (q, J = 6.9 Hz, 8H), 4.67 (s, 8H), 6.75 (s, 8H), 7.52 (s, 4H), 7.56 (s, 2H); ^{13}C NMR (100 MHz, $CDCl_3$) δ 14.2, 21.2, 61.5, 65.6, 113.6, 124.9, 126.0, 128.8, 129.6, 133.7, 136.0, 138.6, 157.0, 169.3; ESI-MS m/z calcd for $C_{64}H_{70}O_{12}N_1$ 1044.4898, found 1044.4890 $(M+H)^+$.

1,3,6,8-Tetrakis(2,6-dimethyl-4-(α-carboxy)methoxyphenyl)pyrene: H_4L

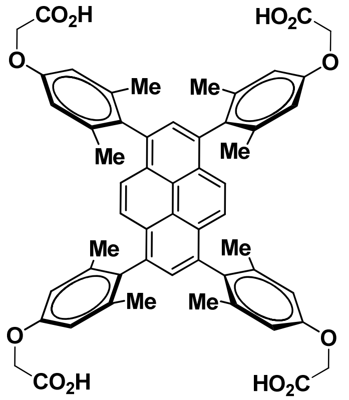

A solution of tetraester **3** (1.0 g, 0.97 mmol) in 30 mL of MeOH was stirred in a round bottom flask with K_2CO_3 (1.62 g, 15.58 mmol) for 12 h. Subsequently, the reaction mixture was stripped off MeOH under vacuo and the resultant solid residue was dissolved in 50 mL of distilled water. This solution was then washed twice with ethyl acetate to remove organic impurities and the aqueous phase was neutralized with 10% aq. HCl, whereby the product precipitated out. The solid material was extracted with ethyl acetate, finally washed with cold distilled water, dried over anhyd Na_2SO_4 and concentrated in vacuo. The crude product was recrystallized using 50% EtOAc/pet. ether to obtain pure **H_4L** as a colorless solid; yield 96% (0.855 g, 0.935 mmol); mp > 300 ºC; IR (KBr) cm^{-1} 2918, 2618, 2593, 1735, 1604, 1475, 1460, 1439, 1380, 1313, 1242, 1195, 1180; ^1H NMR (500 MHz, DMSO-d_6) δ 1.87 (s, 24H), 4.72 (s, 8H), 6.81 (s, 8H), 7.47 (s, 4H), 7.49 (s, 2H); ^{13}C NMR (125 MHz, DMSO-d_6) δ 20.6, 64.4, 113.4, 124.5, 125.2, 128.0, 129.7, 132.1, 135.8, 137.6, 156.9, 170.4; ESI-MS *m/z* calcd for $C_{56}H_{50}O_{12}$ 915.3381, found 915.3381 $[M+H]^+$.

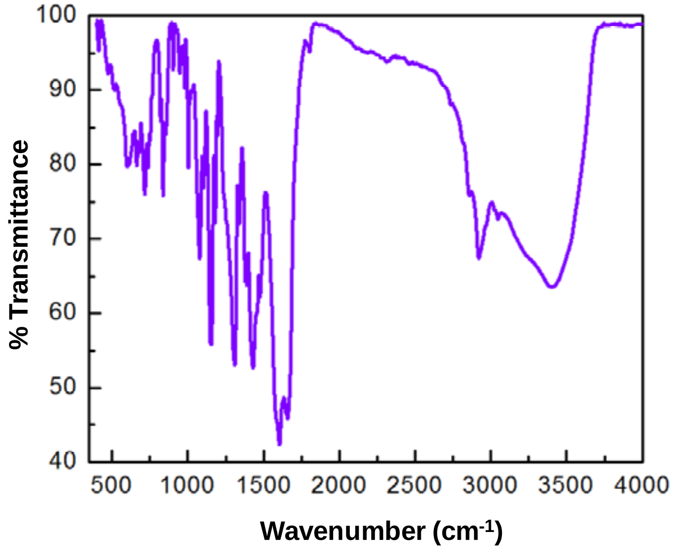

Figure S1. IR spectrum (solid) of **Mn-L**.

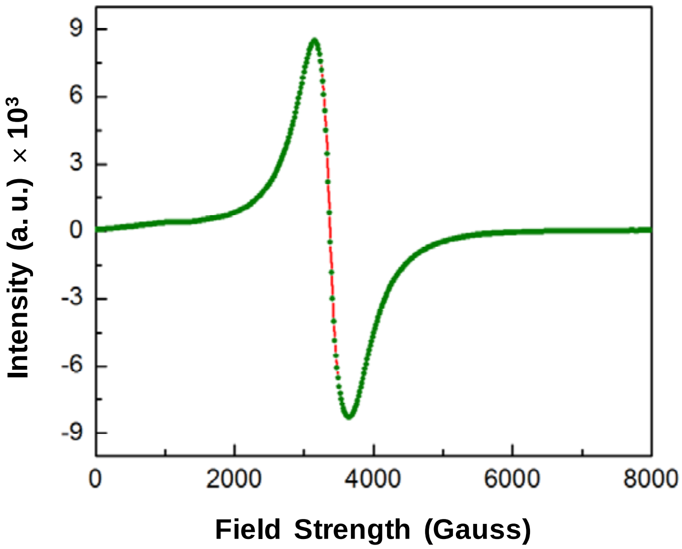

Figure S2. Electron paramagnetic resonance (EPR) spectrum of **Mn-L**.

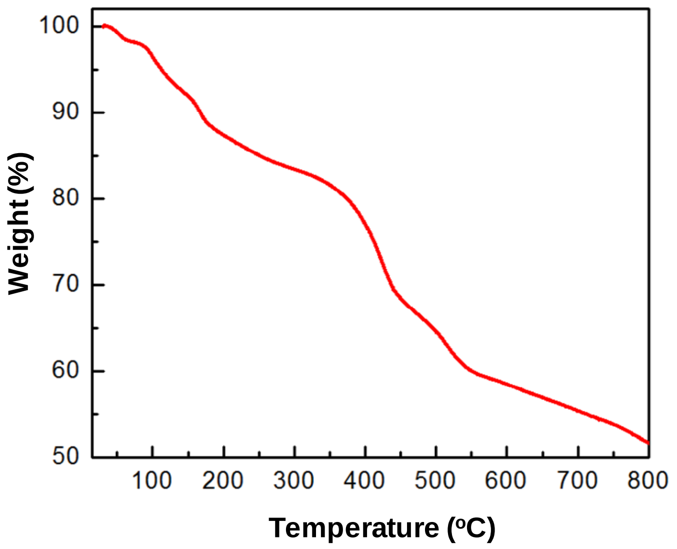

Figure S3. TGA profile of **Mn-L**.

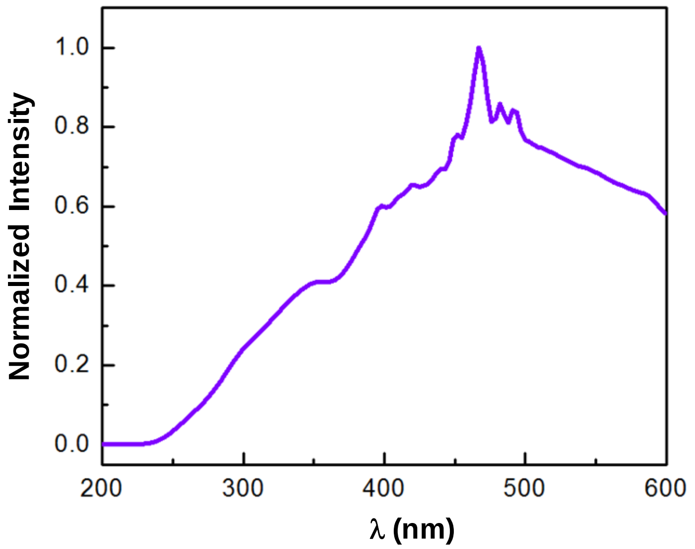

Figure S4. Solid-state excitation spectrum of **Mn-L**.

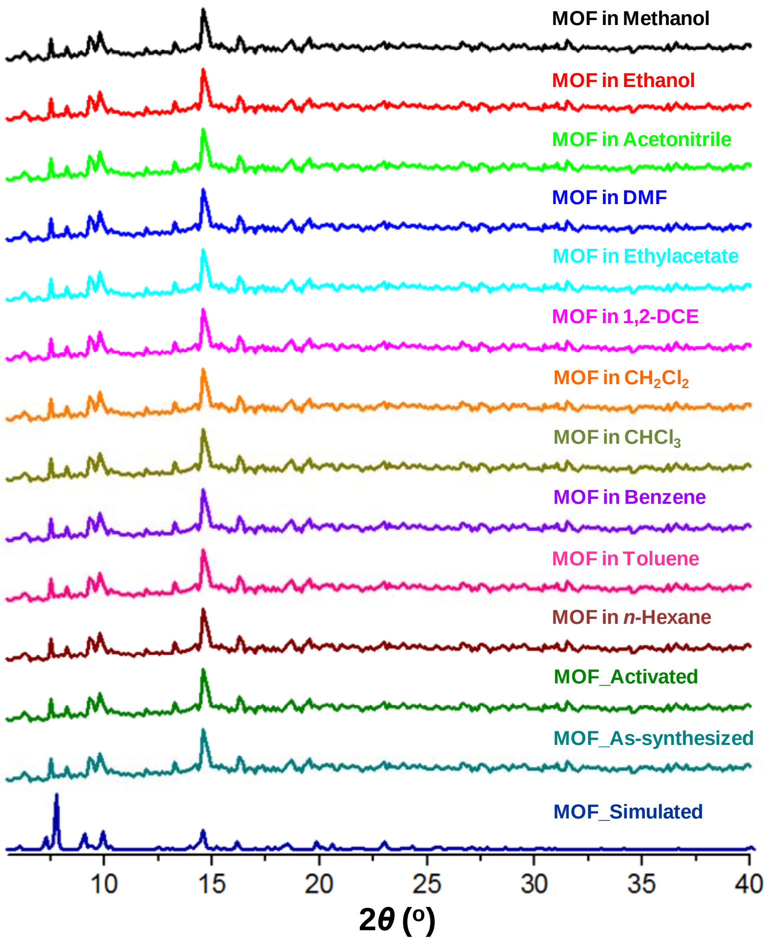

Figure S5. PXRD patterns of variuos solvent-included **Mn-L** crystals.

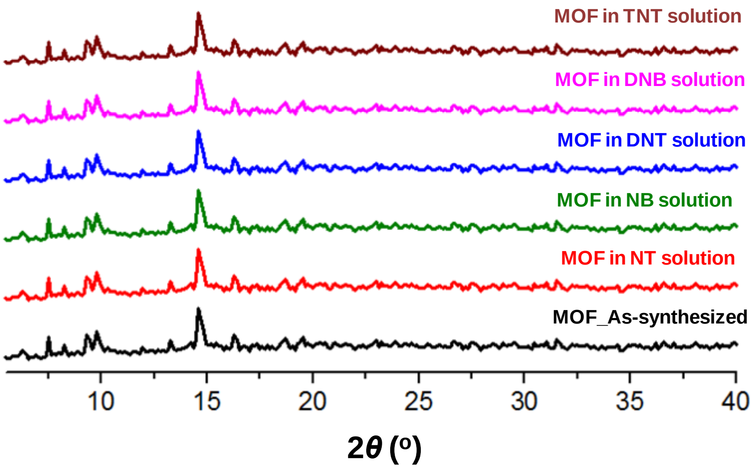

Figure S6. PXRD patterns of **Mn-L** crystals obtained after immersing in solutions of various nitroarmatic analytes in dichloromethane.

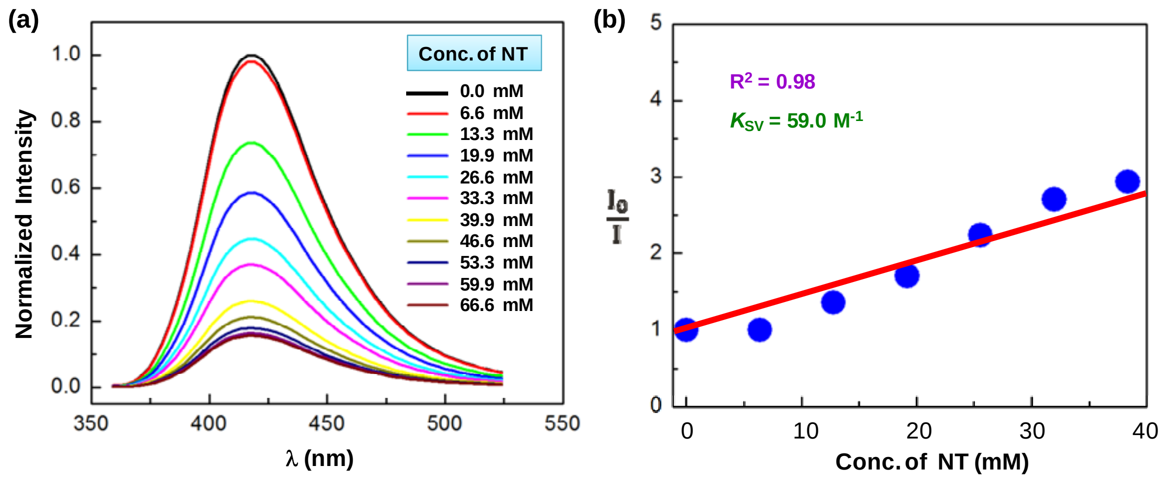

Figure S7. (a) Quenching of fluorescence intensity of **Mn-L** with increasing concentration of NT in DCM (λ_{ex} = 320 nm). (b) Determination of the Stern-Volmer quenching constant.

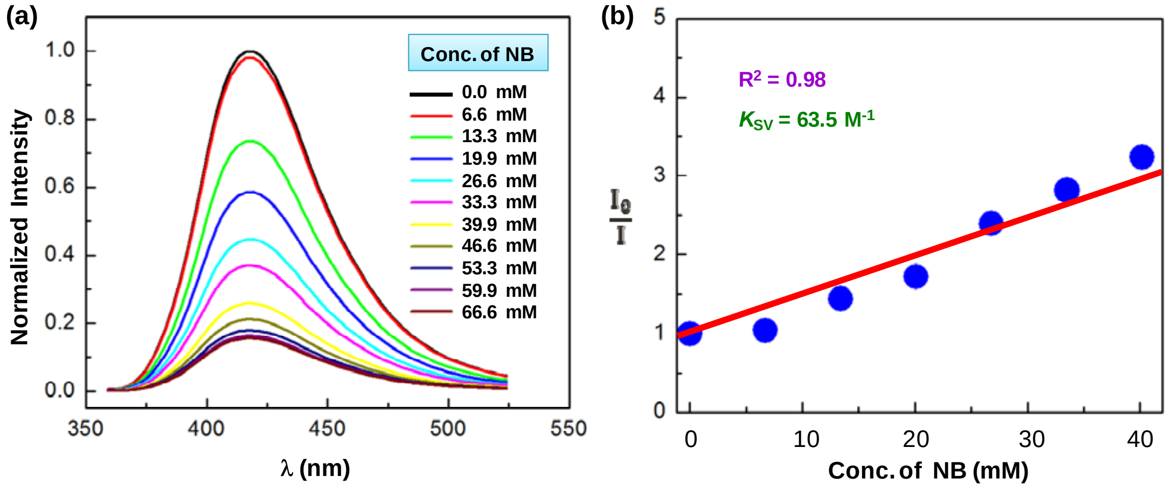

Figure S8. (a) Quenching of fluorescence intensity of **Mn-L** with increasing concentration of NB in DCM (λ_{ex} = 320 nm). (b) Determination of the Stern-Volmer quenching constant.

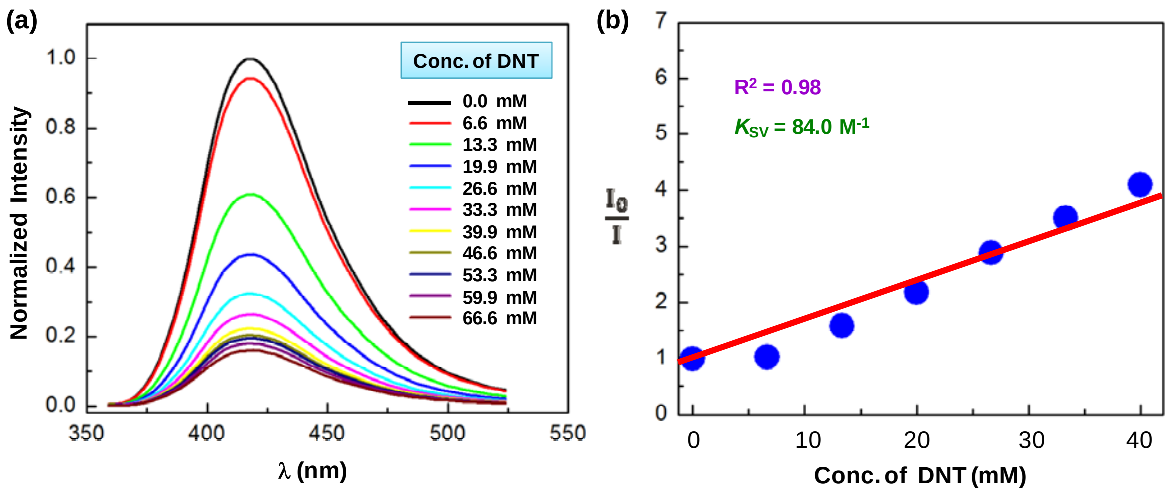

Figure S9. (a) Quenching of fluorescence intensity of **Mn-L** with increasing concentration of DNT in DCM (λ_{ex} = 320 nm). (b) Determination of the Stern-Volmer quenching constant.

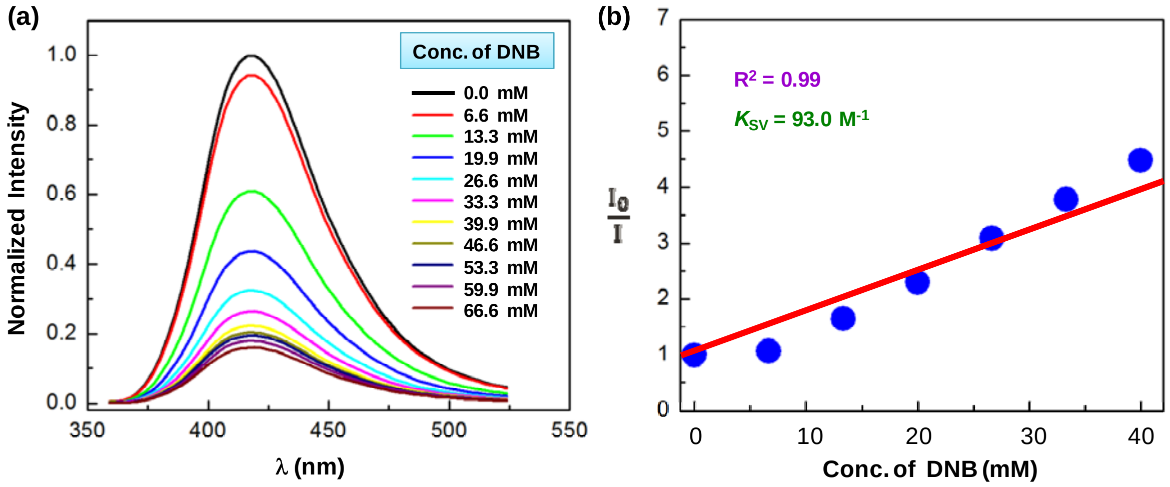

Figure S10. (a) Quenching of fluorescence intensity of **Mn-L** with increasing concentration of DNB in DCM (λ_{ex} = 320 nm). (b) Determination of the Stern-Volmer quenching constant.

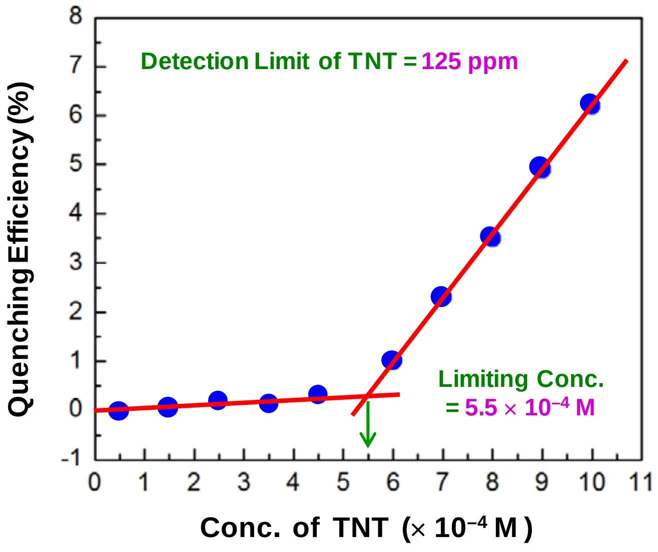

Figure S11. Determination of the sensitivity of **Mn-L** towards the detection of TNT. Notice that the limiting concentration has been calculated from the point of intersection of the two linear fits that are colored in red.

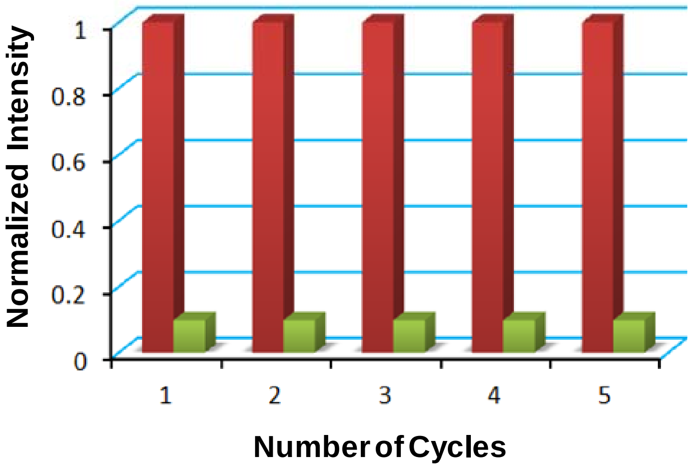

Figure S12. Quenching and recovery test of **Mn-L** with TNT. The red bars represent the original fluorescence intensity of **Mn-L** and the green bars represent the intensity upon addition of 66.6 mM solution of TNT in DCM.

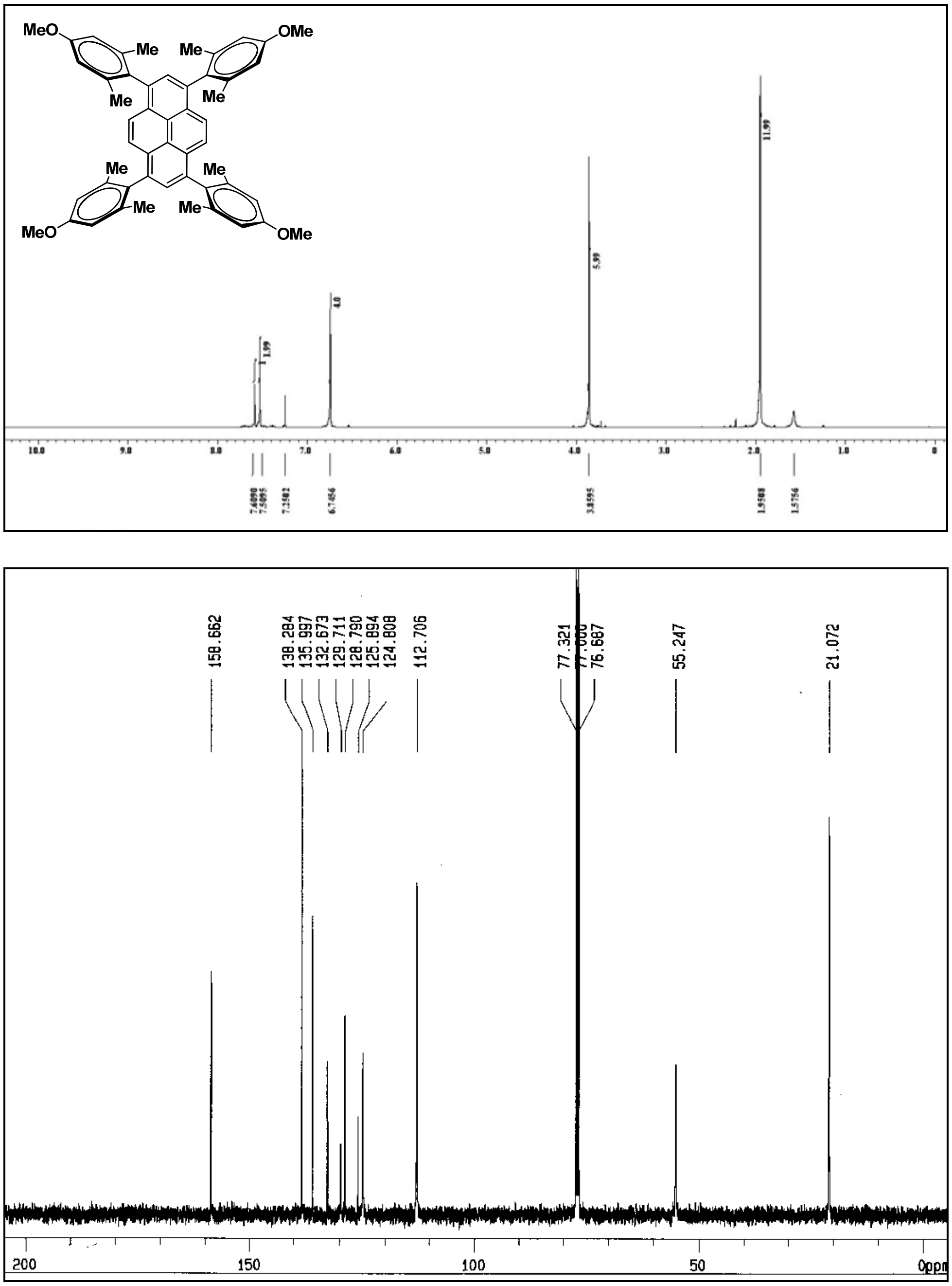

Figure S13. ^{1}H (500 MHz, $CDCl_3$) and ^{13}C NMR (125 MHz, $CDCl_3$) spectra of 1,3,6,8-tetrakis(2,6-dimethyl-4-methoxyphenyl)pyrene.

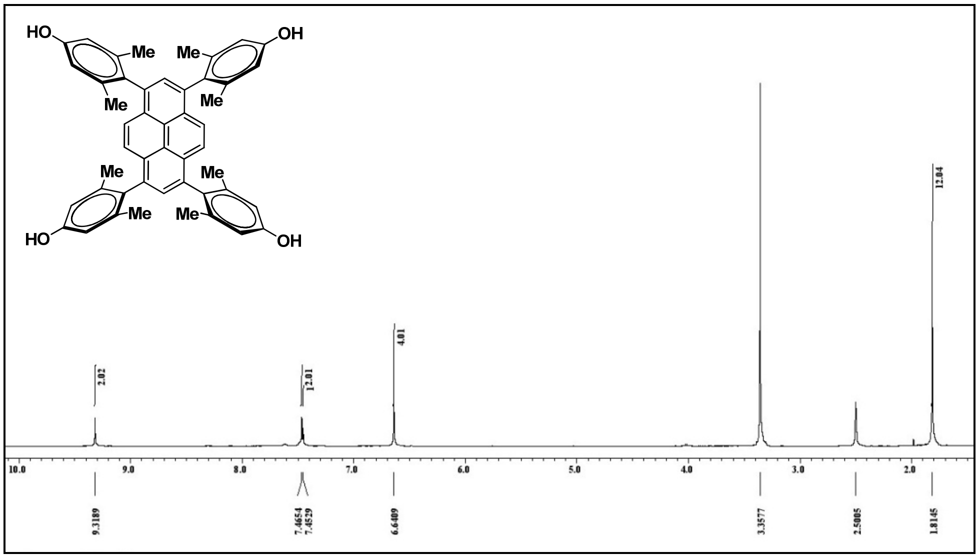

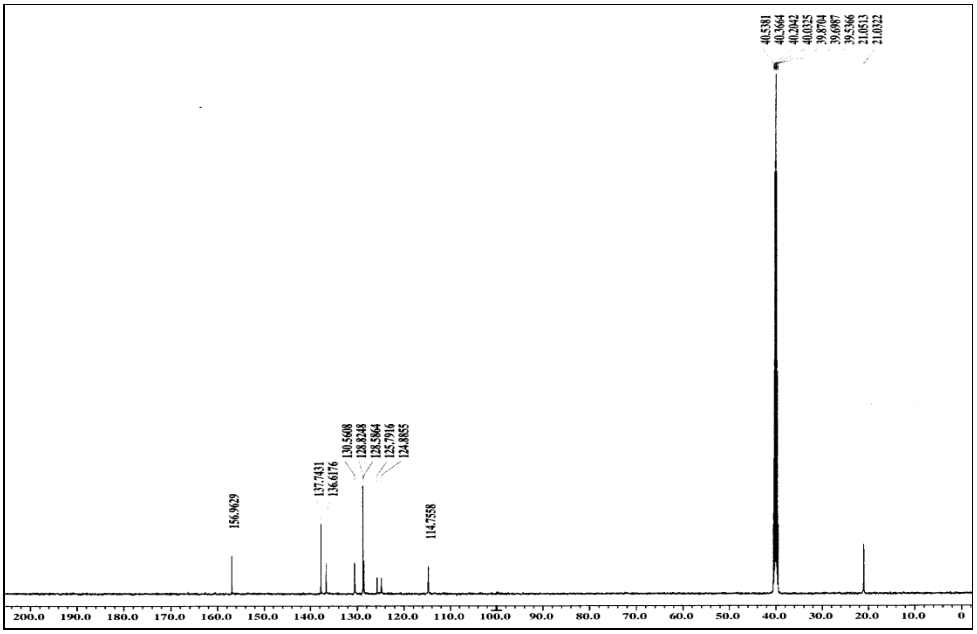

Figure S14. ^{1}H (500 MHz, DMSO-d_6) and ^{13}C NMR (125 MHz, DMSO-d_6) spectra of 1,3,6,8-tetrakis(2,6-dimethyl-4-hydroxyphenyl)pyrene.

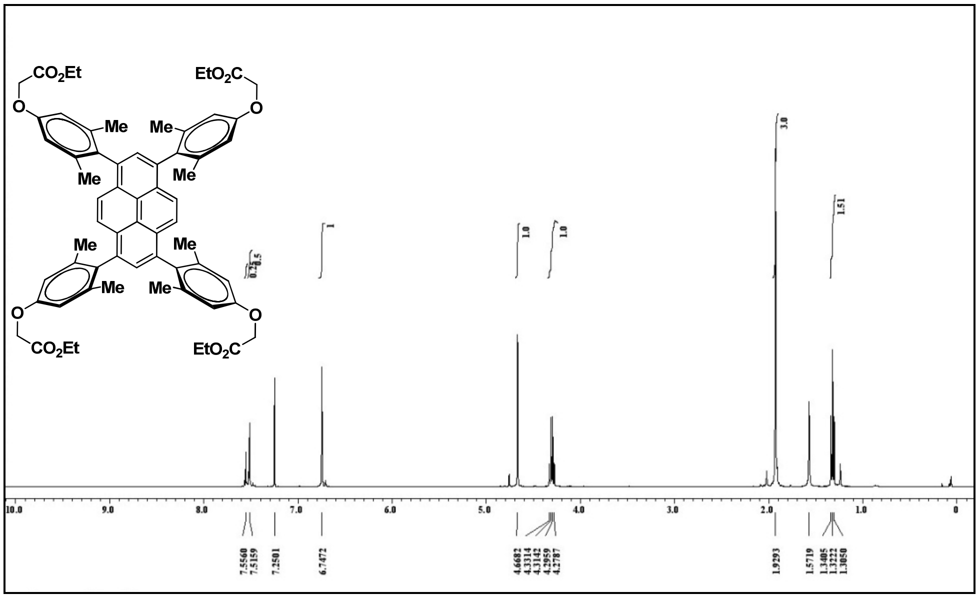

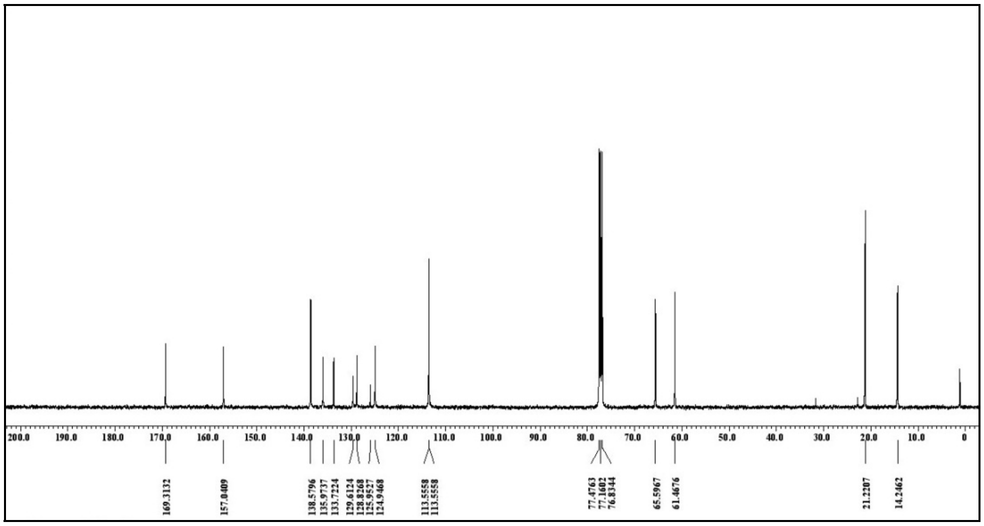

Figure S15. ^{1}H (400 MHz, $CDCl_3$) and ^{13}C NMR (100 MHz, $CDCl_3$) spectra of 1,3,6,8-tetrakis(2,6-dimethyl-4-(α-carboethoxy)methoxyphenyl)pyrene.

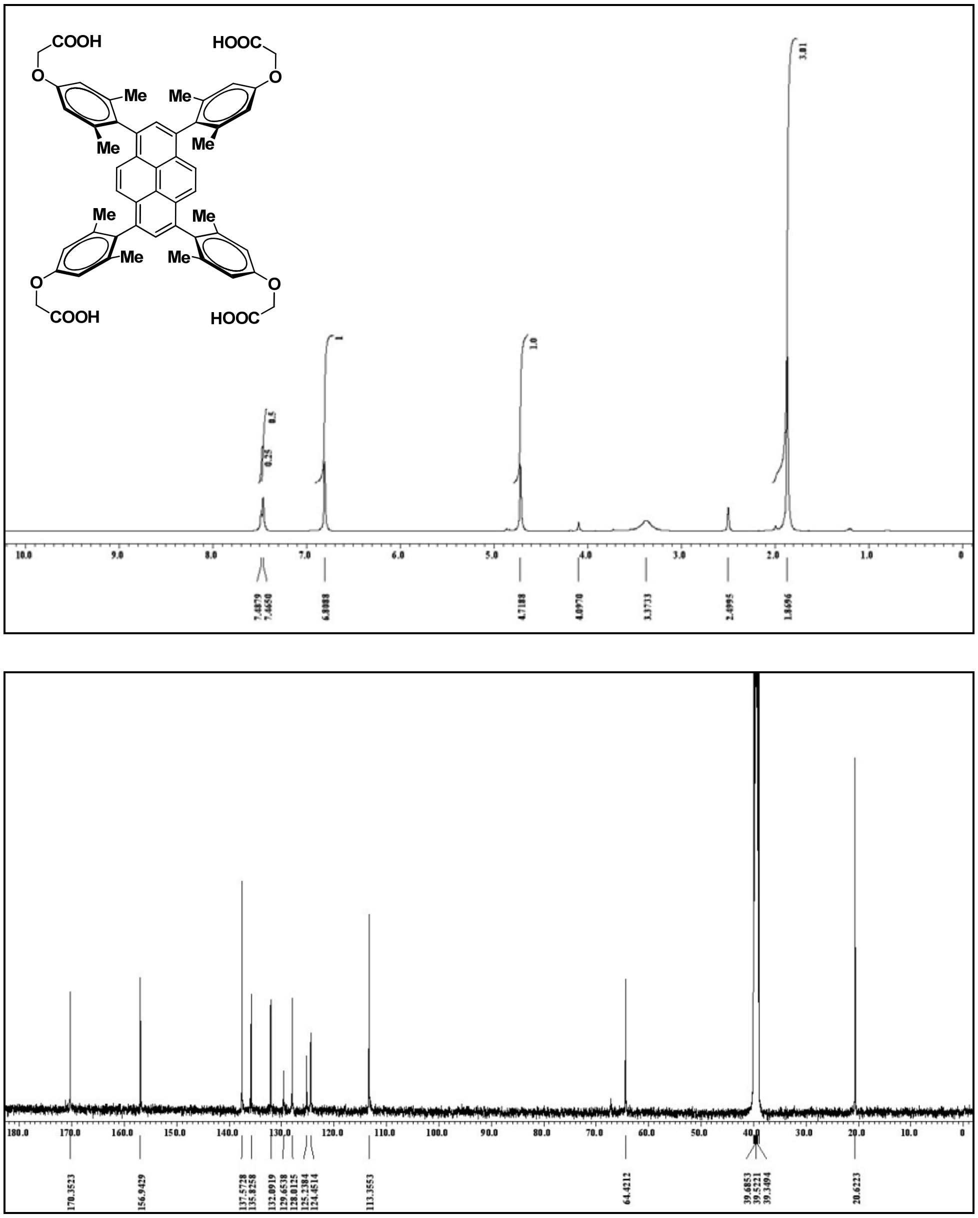

Figure S16. ^{1}H (500 MHz, DMSO-d_6) and ^{13}C NMR (125 MHz, DMSO-d_6) spectra of 1,3,6,8-tetrakis(2,6-dimethyl-4-(α-carboxy)methoxyphenyl)pyrene, $\mathbf{H_4L}$.

checkCIF/PLATON report

Structure factors have been supplied for datablock(s) H4L

THIS REPORT IS FOR GUIDANCE ONLY. IF USED AS PART OF A REVIEW PROCEDURE FOR PUBLICATION, IT SHOULD NOT REPLACE THE EXPERTISE OF AN EXPERIENCED CRYSTALLOGRAPHIC REFEREE.

No syntax errors found. CIF dictionary Interpreting this report

Datablock: H4L

Bond precision: C-C = 0.0067 A Wavelength=0.71073

Cell:	a=19.6786(11)	b=22.7703(13)	c=14.8344(9)
	alpha=90	beta=104.147(1)	gamma=90
Temperature:	100 K		

	Calculated	Reported
Volume	6445.5(6)	6445.5(6)
Space group	P 21/c	P 21/c
Hall group	-P 2ybc	-P 2ybc
Moiety formula	C59 H53 Mn2 N O15, 2(C3 H7 N O)	C59 H53 Mn2 N O15, 2(C3 H7 N O)
Sum formula	C65 H67 Mn2 N3 O17	C65 H67 Mn2 N3 O17
Mr	1272.10	1272.10
Dx,g cm-3	1.311	1.311
Z	4	4
Mu (mm-1)	0.462	0.462
F000	2656.0	2656.0
F000’	2659.97	
h,k,lmax	26,30,19	26,30,19
Nref	16085	16040
Tmin,Tmax	0.912,0.912	
Tmin’	0.912	

Correction method= Not given

Data completeness= 0.997 Theta(max)= 28.337

R(reflections)= 0.1015(8829) wR2(reflections)= 0.2906(16040)

S = 1.046 Npar= 798

The following ALERTS were generated. Each ALERT has the format
test-name_ALERT_alert-type_alert-level.
Click on the hyperlinks for more details of the test.

Alert level A

```
PLAT222_ALERT_3_A Large Non-Solvent   H     Uiso(max)/Uiso(min) ...       10.0 Ratio
```

Alert level B

```
PLAT220_ALERT_2_B Large Non-Solvent   C     Ueq(max)/Ueq(min) Range       10.0 Ratio
PLAT220_ALERT_2_B Large Non-Solvent   O     Ueq(max)/Ueq(min) Range       10.0 Ratio
PLAT230_ALERT_2_B Hirshfeld Test Diff for    O13     --  C58      ..       7.6 su
PLAT242_ALERT_2_B Low         Ueq as Compared to Neighbors for .....       Mn2 Check
PLAT242_ALERT_2_B Low         Ueq as Compared to Neighbors for .....        N3 Check
PLAT430_ALERT_2_B Short Inter D...A Contact  O12     ..  O15      ..      2.74 Ang.
PLAT430_ALERT_2_B Short Inter D...A Contact  O14     ..  O17      ..      2.70 Ang.
```

Alert level C

```
RFACG01_ALERT_3_C  The value of the R factor is > 0.10
            R factor given    0.101
RFACR01_ALERT_3_C  The value of the weighted R factor is > 0.25
            Weighted R factor given    0.291
PLAT084_ALERT_3_C High wR2 Value (i.e. > 0.25) ...................        0.29 Report
PLAT094_ALERT_2_C Ratio of Maximum / Minimum Residual Density ....        2.02 Report
PLAT213_ALERT_2_C Atom O6                 has ADP max/min Ratio .....      3.2 prolat
PLAT213_ALERT_2_C Atom O13                has ADP max/min Ratio .....      3.2 prolat
PLAT213_ALERT_2_C Atom O17                has ADP max/min Ratio .....      3.4 prolat
PLAT213_ALERT_2_C Atom C54                has ADP max/min Ratio .....      3.2 prolat
PLAT213_ALERT_2_C Atom C58                has ADP max/min Ratio .....      3.1 prolat
PLAT214_ALERT_2_C Atom C67      (Anion/Solvent) ADP max/min Ratio          4.6 prolat
PLAT234_ALERT_4_C Large Hirshfeld Difference Mn2      --  O17      ..      0.17 Ang.
PLAT234_ALERT_4_C Large Hirshfeld Difference N3       --  C59      ..      0.22 Ang.
PLAT234_ALERT_4_C Large Hirshfeld Difference N3       --  C60      ..      0.24 Ang.
PLAT241_ALERT_2_C High        Ueq as Compared to Neighbors for .....       O13 Check
PLAT241_ALERT_2_C High        Ueq as Compared to Neighbors for .....       C58 Check
PLAT243_ALERT_4_C High  'Solvent' Ueq as Compared to Neighbors of          C67 Check
PLAT244_ALERT_4_C Low   'Solvent' Ueq as Compared to Neighbors of           N2 Check
PLAT244_ALERT_4_C Low   'Solvent' Ueq as Compared to Neighbors of           N1 Check
PLAT341_ALERT_3_C Low Bond Precision on  C-C Bonds ...............      0.0067 Ang.
PLAT412_ALERT_2_C Short Intra XH3 .. XHn      H59A   ..  H60C     ..      1.88 Ang.
PLAT601_ALERT_2_C Structure Contains Solvent Accessible VOIDS of .          84 Ang3
```

Alert level G

```
PLAT004_ALERT_5_G Polymeric Structure Found with Maximum Dimension           3 Info
PLAT072_ALERT_2_G SHELXL First  Parameter in WGHT Unusually Large.        0.16 Report
PLAT232_ALERT_2_G Hirshfeld Test Diff (M-X)  Mn2     --  O2       ..       8.5 su
PLAT804_ALERT_5_G Number of ARU-Code Packing Problem(s) in PLATON            7 Info
```

```
  1 ALERT level A = Most likely a serious problem - resolve or explain
  7 ALERT level B = A potentially serious problem, consider carefully
 21 ALERT level C = Check. Ensure it is not caused by an omission or oversight
  4 ALERT level G = General information/check it is not something unexpected

  0 ALERT type 1 CIF construction/syntax error, inconsistent or missing data
 20 ALERT type 2 Indicator that the structure model may be wrong or deficient
  5 ALERT type 3 Indicator that the structure quality may be low
  6 ALERT type 4 Improvement, methodology, query or suggestion
  2 ALERT type 5 Informative message, check
```

It is advisable to attempt to resolve as many as possible of the alerts in all categories. Often the minor alerts point to easily fixed oversights, errors and omissions in your CIF or refinement strategy, so attention to these fine details can be worthwhile. In order to resolve some of the more serious problems it may be necessary to carry out additional measurements or structure refinements. However, the purpose of your study may justify the reported deviations and the more serious of these should normally be commented upon in the discussion or experimental section of a paper or in the "special_details" fields of the CIF. checkCIF was carefully designed to identify outliers and unusual parameters, but every test has its limitations and alerts that are not important in a particular case may appear. Conversely, the absence of alerts does not guarantee there are no aspects of the results needing attention. It is up to the individual to critically assess their own results and, if necessary, seek expert advice.

Publication of your CIF in IUCr journals

A basic structural check has been run on your CIF. These basic checks will be run on all CIFs submitted for publication in IUCr journals (*Acta Crystallographica*, *Journal of Applied Crystallography*, *Journal of Synchrotron Radiation*); however, if you intend to submit to *Acta Crystallographica Section C* or *E*, you should make sure that full publication checks are run on the final version of your CIF prior to submission.

Publication of your CIF in other journals

Please refer to the *Notes for Authors* of the relevant journal for any special instructions relating to CIF submission.

PLATON version of 29/01/2015; check.def file version of 29/01/2015

Datablock H4L - ellipsoid plot

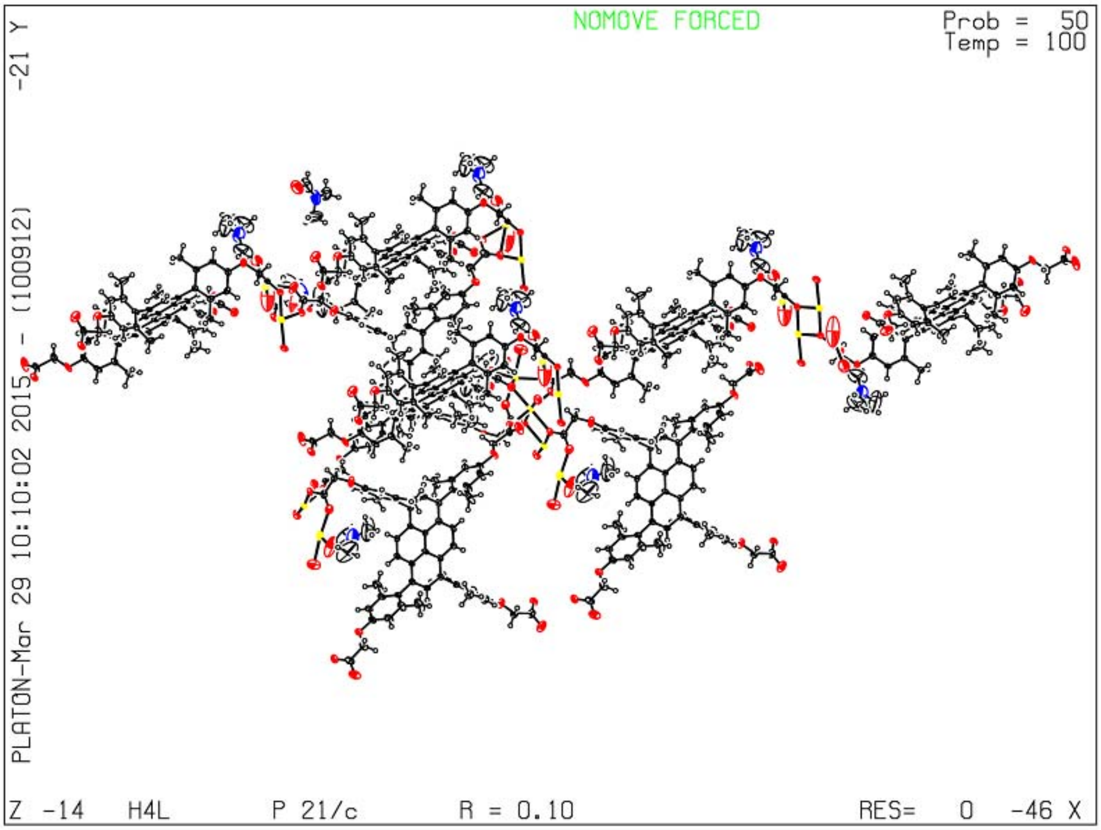

Explanation for Checkcif

Check cif	Explanation
Alert A, B, C, G	Large parameter shift to su ratio due to COOH groups in non-ideal geometry. Crystal has a polymeric structure with large void volume.
